# An evaluation of the performance characteristics of SCR utilizing a Fe_2_O_3_–SiO_2_/Al_2_O_3_ synthesized catalyst for effective diesel engine exhaust emission reduction

**DOI:** 10.1038/s41598-026-43472-1

**Published:** 2026-03-17

**Authors:** S. Premkumar, S. Panneerselvam, Dhinesh Balasubramanian, Utku Kale, Artūras Kilikevičius

**Affiliations:** 1 Center for Sustainable Materials and Surface Metamorphosis, Chennai Institute of Technology, Chennai, 600069 India; 2 Department of Mechanical Engineering, Faculty of Engineering and Technology, SRM Institute of Science and Technology, Kattankulathur, Chennai, India; 3 Department of Port Engineering, Lithuanian Maritime Academy (LMA), Vilnius Gediminas Technical University, Klaipėda, Lithuania; 4 Department of Aeronautics and Naval Architecture, Faculty of Transportation Engineering and Vehicle Engineering, Budapest University of Technology and Economics, Műegyetem Rkp. 3., Budapest, 1111 Hungary; 5 Mechanical Science Institute, Vilnius Gediminas Technical University, Plytinės G. 25, 10105 Vilnius, Lithuania

**Keywords:** Diesel engine, SCR, Fe_2_O_3_–SiO_2_/Al_2_O_3_ catalyst, Exhaust Emission reduction, Synthetic catalytic performance

## Abstract

Selective Catalytic Reduction (SCR) helps to reduce Nitrogen Oxide (NO) emissions from diesel engines using catalytic materials made of thermally stable non-precious metals. A dual support SCR catalyst using iron oxide (Fe_2_O_3_) and silica/alumina (SiO_2_/Al_2_O_3_) has developed from low-cost materials. This catalyst has a monolith design for application to engines at full scale. The dual support provides a high level of interface between the iron oxide and silica/alumina that allows higher iron loadings, better iron distribution, and greater thermal stability of the Fe^3+^/Fe^2+^ redox couples across a wide temperature range (150–600 °C). Analytical techniques like (X-ray Diffraction (XRD), Scanning Electron Microscopy (SEM), Brunauer–Emmett–Teller Surface Area (BET) and Fourier Transform Infrared (FTIR) Spectroscopy confirmed the presence of a thermally stable and mesoporous catalyst with an even distribution of Lewis and Bronsted acid sites. Industrial applications of this catalyst have been performed on a diesel engine fuel as a diesel and diesel plastic oil blend (DPB) (B50) with a power output of 5.2 kW and significant conversion rates of 85% of NO at maximum load, and Hydrocarbons (HC), Carbon Monoxide (CO) and smoke opacity have been reduced by 65%, 55%, and 60%, respectively. Statistical analysis of results across all experimental conditions, including ANOVA, was conducted to establish the reproducibility of the findings. This catalyst exhibited consistent activity when tested with conventional diesel fuel as well as blended fuels composed of diesel and post-industrial waste. No precious metals or polymers have been used in the synthesis of the dual support SCR catalyst or subsequent formulations for commercial application. Overall, the results demonstrated that low-cost precursors could serve as effective catalysts for reducing NO emissions from diesel engines.

## Introduction

Diesel engines are widely used in many industries, i.e., transportation and agriculture, and still generate a high amount of NO_x_, HC, CO, and particulate matter because they have high thermal efficiency and durability^1^. It resulted in strict emission limits imposed by regulatory agencies, such as the Euro VI and Bharat Stage VI standards. These have led to the rapid development of more effective technologies for controlling emissions through exhaust after-treatment^2^.

Additionally, plastic waste continues to accumulate globally (over 400 million tons a year), with less than 10% of this material have recycled^3,4^, there is a growing recognition that catalytic pyrolysis provides to create a circular economy for converting plastics back into liquid HCs. that can be used for fuels. The liquid fuels produced from plastic pyrolysis have combustion properties similar to those of diesel fuels, which will allow partial replacement of fossil fuels while simultaneously managing some of the challenges associated with plastic waste management^5–8^. However, when plastics or plastic pyrolysis oil are used as a fuel blend with diesel, it often creates higher levels of NO_x_ and unburned emissions than when diesel is used alone^9–11^. Because of this, post-combustion emissions control systems are needed to reduce these elevated levels of NO_x_ and unburned HC^12^.

SCR is currently documented as the most efficient means of reducing NO_x_ emissions in diesel engines that operate with high oxygen levels^13–16^. While currently deployed vanadium and Platinum Group of Metal (PGM) based SCR catalysts are the most productive catalyst types currently known to remove NO_x_ under high oxygen conditions, they are also the most prohibitively expensive, toxic, sulfur sensitive, and thermally unstable^17–22^. Non-precious metal catalysts, in particular, and Fe_2_O_3_based SCR catalysts are gaining attention due to their comparatively lower cost, better environmental profile, and higher availability in the environment than precious metals^23–27^. Further to enhance the performance of Fe_2_O_3-_based SCR catalysts SiO_2_-Al_2_O_3_-based support materials allow for improving thermal stability and surface acid properties thus reducing the potential for sintering and improving the broad operating temperature of Fe_2_O_3_-based SCR catalysts^28–31^.

**Fig. 1:**
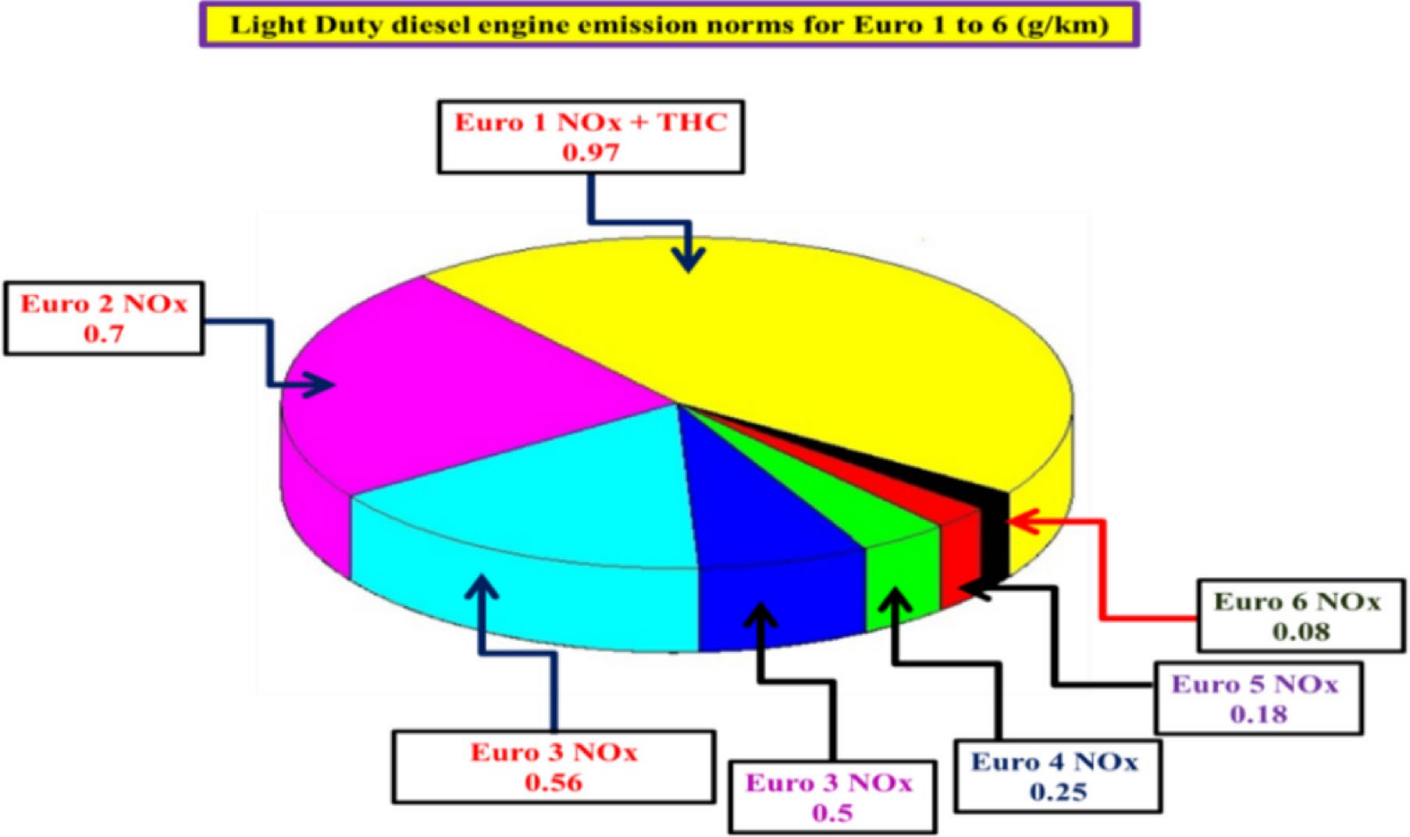
NO_x_ emissions limits enforced by EU regulations for diesel light-duty vehicles (LDVs)^38^.

**Fig. 2:**
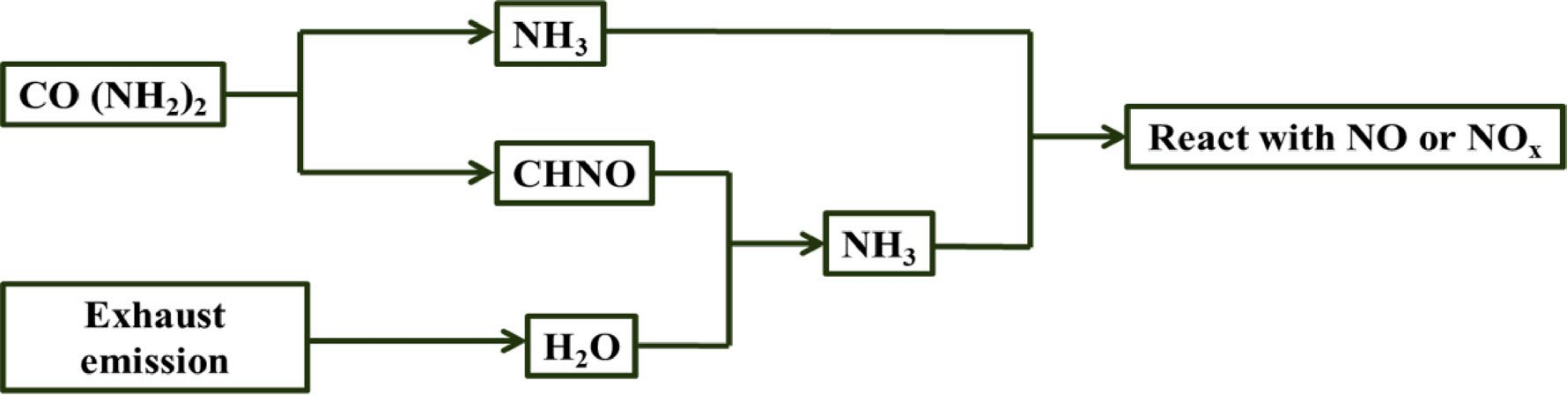
An overview of urea decomposition reacts during ammonia production^39^.

Although the benefits of Fe_2_O_3_based SCR catalysts have been demonstrated which have limited number of studies that examine the use of Fe_2_O_3_based SCR catalysts in a monolithic honeycomb configuration and their potential use in diesel engine emissions’ performance within the context of diesel engine exhaust emissions originating from diesel engines operating on diesel fuels and plastic-derived pyrolysis fuel blends. To strengthen the current study of knowledge regarding the use of Fe_2_O_3_-based SCR catalysts in a diesel engine setting, the present study evaluates the use of a Fe_2_O_3_–SiO_2_/Al_2_O_3_honeycomb SCR catalyst during operation of diesel engines fueled with diesel and plastic-derived pyrolysis oil fuel blends and examines both thermal stability and real-world performance for diesel engine NO_x_ emissions^32–36^. The use of SCR catalysts in the diesel exhaust system is a fundamental part of modern emission control systems^37^. Combining the SCR unit(s) with Diesel Oxidation Catalyst (DOCs) and Diesel Particulate Filter (DPFs) allows the use of a sequential approach to treat NO, HCs, CO, and PM simultaneously^38^. The Fe_2_O_3_–SiO_2_/Al_2_O_3_catalyst is well-suited for application of these types of comprehensive systems and provides high oxidative capability as well as high thermal stability. The European Union emission standards for Light Duty Diesel Vehicles (LDVs) have been summarized in Fig. 1, as well as the ability of these vehicles to control NO emissions. The conversion of urea to ammonia through thermal decomposition takes place during the SCR stage of the process. A depiction of the ammonia production can be seen on Fig. 2. A crucial aspect of the SCR process is that, by oxidizing CO and HCs at the same time while reducing NO_x_, the total activity of the SCR unit has increased, thus preventing ammonia slip and secondary emissions^39^.

The growing interest in sustainable mobility and circular energy systems has prompted the investigation of a new way to reduce emissions with a combined approach which makes use of pyrolysis derived from the decomposition of plastic waste. One of the replacement fuels for diesel in an integrated waste to energy system and the addition of an advanced catalytic treatment system attached to the vehicle using that type of fuel. This combined technology, when utilized in conjunction with existing technologies such as plastics, means reduced dependency on fossil fuels and less opportunity for plastic pollution in our environment. The advantages of these combinations of technologies also align with the UN Sustainable Development Goals (SDG 7 represents as Affordable and clean energy, SDG 11 represents as Sustainable Cities and Communities and SDG 13—Climate Action) and have become an integral part of the development of catalytic systems to use in a range of applications. As market demands for environmentally friendly solutions and the need for the reduction of greenhouse gasses increase, advantages of these technologies will become important support mechanisms for emission reductions for a significant portion of low-cost vehicles. The standards of emission mentioned in Table 1^40^. Also support this assertion.

**Table 1:** Regulated emission limits for diesel engines^6^.

Emission standards	NO (g/kWh)	HC (g/kWh)	CO (g/kWh)	Smoke (g/kWh)
Euro 1	8.0	1.1	4.5	0.8
Euro 2	7.0	1.1	4.0	0.5
Euro 3	5.0	0.66	2.1	0.5
Euro 4	3.5	0.46	1.5	0.2
Euro 5	2.0	0.46	1.5	0.2
Euro 6	0.4	0.13	0.5	0.02
Euro 7	0.2	0.05	0.2	0.01

Unlike standard Fe/SiO_2_ and Fe/Al_2_O_3_ catalytic systems that require washing or powdering are generally supported by an inert ceramic substrate. The present work describes a self-supported catalytic mold comprised of a monolithic formulation of Fe_2_O_3_, SiO_2_ and Al_2_O_3_, which functions as both a load-bearing structure and an effective catalyst. The results of the current study indicate the substrate-less arrangement results in greater contact and thus interaction between the active site of the catalyst; the Fe–O–Si and Fe–O–Al interfaces and Fe ions were more evenly distributed on the surface as well, stabilizing the surface acidity for NH_3_ absorption while reducing Fe particle growth as a result of elevated outlet gas temperatures.

To specifically compare the performance of the proposed technology with that of commercially available technologies, a quantitative analysis of the performance of selected commercially available catalyst systems (Fe/Al_2_O_3_, Fe/SiO_2_, V_2_O_5_–WO_3_/TiO_2_ and noble metals in SCR applications) was performed for similar operating conditions, with the resultant data presented in Table 2. Fe-based catalyst systems typically convert 50–65% of the available NO_x_ in the narrow range of 250–550 °C, while the vanadium oxide-based systems convert substantially more however result in toxicity. It has limited thermal stability/flexibility compared to Fe-based catalysts. Conversely, the Fe_2_O_3_–SiO_2_–Al_2_O_3_ catalyst from the current study demonstrated the convert of 80–85% NO, 55–65% HC and 45–55% CO across a continuous temperature range of 150–600 °C.

**Table 2:** Quantitative evaluation and benchmarking metrics.

Types of catalyst	Catalyst configuration	Temperature range	NO conversion (%)	HC conversion (%)	CO conversion (%)	Key limitation	Referencess
Fe/Al_2_O_3_	Fe oxide wash coated on γ-Al_2_O_3_ honeycomb	250 -500	55–65	45–55	40–55	Fe sintering above 500 °C; reduced NH_3_ storage at low temperature	^16^
Fe/SiO_2_	Fe oxide dispersed on SiO_2_	300–550	50–60	40–50	35–50	Weak surface acidity; limited low-temperature SCR activity	^15^
V_2_O_5_-WO_3_/TiO_2_	Vanadium based catalyst on TiO_2_	300–450	85–95	55–65	50–60	Narrow temperature window; vanadium toxicity; sulphur sensitivity	Commercial SCR
PGM based (Pt, Pd, Rh)	Nobel metal	200–600	< 70 lean SCR	85–95	90–95	Very high cost; sulphur poisoning; poor lean-NOₓ selectivity	Automotive DOC/SCR
Present work – Fe_2_O_3_-SiO_2_/Al_2_O_3_ (mold)	Self -supporting Fe_2_O_3_–SiO_2_/Al_2_O^3^ monolithic catalyst	150–600	80–85	74	60	Long-term ageing and extended sulphur durability require further study	Current study

Another significant innovation is the SCR tested catalyst at the engine level, under actual diesel exhaust conditions, an approach that has not been widely discussed for SCR systems using Fe–Si–Al. The catalyst adapted to operate during all load conditions in the exhaust manifold of a 5.2 kW single-cylinder diesel engine, and the structural and chemical stability were characterized by FTIR, XRD, and SEM. It analyzes fresh, exhaust-exposed, and post-reaction catalysts, resulting in direct evidence of structure–activity relationships under realistic SCR operating conditions.

In conclusion, this study indicates the presence of a self-supporting monolithic SCR catalyst based on Fe, which contributes to enhanced SCR performance over the incumbent systems with respect to both temperature range and performance. The new SCR system will complement the existing SCR systems and provide an alternative source for diesel compatibility and scalability.

The current state of SCR technology presents significant limitations. The most common type of catalyst used nowadays is based on noble metals (e.g. platinum or palladium). These catalysts are highly active; however, they are also very expensive, sensitive to sulfur poisoning, and possess a tendency to experience thermal sintering. The second main form of SCR catalyst is the vanadium-based catalyst, which is limited in operation by its narrow catalyst temperature range. Additionally, vanadium-based SCR catalysts raise concerns regarding toxic byproducts and durability in hydrothermal environments. The third main form of SCR catalyst is the zeolite-based SCR catalyst, which is effective at low temperatures but suffers from four main types of limitations: water poisoning, limited high-temperature stability, very complex synthesis routes, and ultimately widespread deployment limitations due to cost. As a result, it needs further development of inexpensive, thermally stable, and environmentally friendly SCR catalysts which are capable of effectively reducing NO_x_ emissions over a wide range of diesel exhaust temperatures (150–600 °C) and under realistic diesel engine conditions. The multi-component Fe_2_O_3_–SiO_2_/Al_2_O_3_ approach has proven to be a promising solution; however, it is a relatively unstudied area of research that can provide an innovative solution by combining the redox activity of Fe_2_O_3_ with the thermal stability and surface acidity of both the SiO_2_ and Al_2_O_3_ supports. There are very limited studies currently evaluating these types of multi-component systems in monolithic form using diesel exhaust under realistic diesel engine operating conditions and, in particular, using plastic derived and blended fuels with high aromatic content, increased NO_x_ and unburned emissions.

Although Fe-based SCR catalysts studied extensively, the development and validation of low-cost Fe_2_O_3_–SiO_2_/Al_2_O_3_ composites fabricated into monolithic honeycomb shapes have received relatively little attention particularly regarding real diesel exhausts from plastic-derived fuels. Because of the lack of systematic studies, the usefulness, lifecycle and emissions control of these composites cannot be realistically assessed.

### Comparison with commercial SCR catalysts

To evaluate the feasibility of using a catalyst that is less expensive, thermally stable, and environmentally friendly for controlling diesel exhaust emissions, the performance of the synthesized Fe_2_O_3_–SiO_2_/Al_2_O_3_ catalyst was compared with commercially available PGM and V_2_O_5_based SCR catalysts.

While the PGM-based SCR catalysts perform well at low temperatures, they have limitations such as high material costs, susceptibility to sulfur poisoning, and thermal sintering due to prolonged exposure to high-temperature exhaust gases. The Fe_2_O_3_–SiO_2_/Al_2_O_3_ catalyst compares favorably with regard to NO conversion efficiency in the medium to high temperature range (300–450 °C) and it has improved thermal stability and deactivation resistance approximately similar to PGM SCR catalysts, thus making it applicable for usage in most diesel engine operating conditions.

Although the V_2_O_5_–WO_3_/TiO_2_ catalysts are the most widely used type of SCR catalyst in stationary SCR applications, their use is restricted by the potential for vanadium toxicity and a narrower operating temperature range than is currently available. Another concern with V_2_O_5_–WO_3_/TiO_2_ catalysts is the possibility of N_2_O forming under elevated temperature conditions. The Fe-based catalyst can provide an alternative solution because it has an environmentally benign composition and a much wider operating temperature window, as well as stable redox properties. The dual-support hybrid structure generated from natural sand and waste fly ash enables a more uniform dispersion of Fe and surface acidity, providing excellent SCR catalytic activity without the need to use precious metals or hazardous materials.

Additionally, the formation of a monolithic honeycomb design allows for direct incorporation within diesel exhaust systems due to its low pressure drop and excellent structural integrity. Catalysts tested in an operational diesel engine running on diesel fuel or blends of gasoline and diesel fuel demonstrated that these catalysts could be viable, sustainable and scalable alternatives to conventional SCR catalysts. A comparison of SCR catalyst systems is provided in Table 3.

**Table 3:** Performance comparison of SCR catalyst systems.

Parameter	PGM Based catalyst	V_2_O_5_ based Catalyst in SCR technique	Fe_2_O_3_-SiO_2_/Al_2_O_3_ present catalyst
Active phase	Pt, Pd and Rh	V_2_O_5_–WO_3_/TiO_2_	Fe_2_O_3_
Material cost	Very high	Moderate	Low
Environmental concern	Noble metal extraction	Vanadium toxicity	Environmentally benign
Typical operating temperature (°C)	200–350	300–400	300–450
Thermal stability	Limited at high temperatures	Moderate	High
Sulphur tolerance	Limited at high temperature	Moderate	Good
Risk of N_2_O formation	Low	Moderate—high	Low
Catalyst structure	Monolith	Monolith	Mold
Fuel types	Diesel	Diesel	DPB
Applicability of mobile engines	Cost limited	Limited	High
Sustainability	Low	Moderate	High

The objective of this research study is to develop the next wave of catalytic systems to use in hybrid fuels and circular carbon economies through a collaborative interdisciplinary effort involving applied combustion engineering and materials sciences and technology. It is anticipated that the present research will enhance the understanding of structure–activity relationships in multiple component oxide catalysts, allowing for the development of emissions control devices which will further drive the adoption of sustainable and clean combustion practices and emissions.

Previous research has documented that PGMs are widely used in diesel engine vehicle exhaust systems because of their effectiveness in decreasing exhaust emissions (refer to Table 3). Commonly, PGMs are applied to either ceramic substrate materials or metal catalytic converter systems, typically configured as monolithic honeycomb structures, as described above. Other previous research has investigated the efficiency of PGM catalysts in comparison to traditional catalysts and reported their ability to operate efficiently and effectively at very low temperatures. This work describes a unique as well as highly efficient bi-functional catalytic system which consists of an iron oxide-silicon catalyst and is constructed using a mold similar in shape to the honeycomb structures typically associated with honeycomb design. The use of the metal improves the efficiency in emission reduction, while the synthesis of these catalysts will also provide better control over the performance of the final product.

## Materials and methods

### Materials

Plastics oil with high calorie content is purchased from M/s. Rathi Rubber Private Limited in Chennai. The chemical composition of plastic oil is composed of dibenzenic propane-based HC and is almost identical to pure diesel. Chemicals, which include 98% sulfuric acid and 0.01M hydrochloric acid, used as solvents, can be obtained from SRL Chemicals in India.

### Plastic oil process

We studied the physical and chemical properties of plastic oil. The plastic oil was equally combined with diesel in order to make B50 as DPB. The chemical makeup and properties of plastic oil, particularly DPB, are very much alike to pure diesel. Properties of the fuels that we studied, including diesel, plastic oil and B50, are shown in Table 4. The controlled combustion process starts with converting plastic waste to oil and taking away oxygen. As plastic waste melts and breaks down, the polymers convert into small molecules (mass fractures). These smaller molecules are then converted into oligomers and can again bond together to make monomers. The degree of decomposition can be affected by different factors including temperature, residence time, and synthesis catalyst. Pyrolysis can be done with or without a catalyst. Therefore, both thermal and catalytic pyrolysis will be conducted. This occurs because most of the waste plastic is converted to fuel; the plastic will eventually become plastic oil mixed with diesel. We confirmed the consistency of the data by measuring and recording three measurements and considered all three to be valid based on the two percent difference or less.

**Table 4:** Properties of test fuels determined using standard methods^6^.

Properties	Equipment	Test method	Test frequency	Diesel	Waste plastic oil	DPB (Diesel plastic blend)	Final report
Calorific value (kJ/kg)	Bomb calorimeter	ASTM D240	3 times (n = 3)	44,460	42,325	43,555	Mean ± SD
Flash point (°C)	Pensky – Martens closed cup tester	ASTM D93	3 times (n = 3)	50	42	46	Mean ± SD
Kinematic viscosity (Cst) 40°C	Redwood viscometer	ASTM D 445	3 times (n = 3)	2.12	2.50	2.3	Mean ± SD
Density (kg/m^3^)	Digital densitometer	ASTM D4052	3 times (n = 3)	832	793	797	Mean ± SD
Cetane number	Ignition quality tester	ASTM D 613	3 times (n = 3)	54.28	40.02	47.28	Mean ± SD

### Preparation of synthesis catalyst—Fe_2_O_3_-SiO_2_/Al_2_O_3_ based mold

The sources of aluminosilicate were coal-based fly ash (approximately 55 wt. % SiO_2_ and approximately 28 wt% Al_2_O_3_) and beach sand (approximately 92 wt% SiO_2_, approximately 3 wt% Fe_2_O_3_, and approximately 2 wt% Al_2_O_3_). The raw sand was washed three times (with 1.5 L distilled water for each wash). The washed sand was allowed to settle for six hours after each of the three washings remove soluble impurities. The washed sand was then dried at 110°C for twelve hours, ground, and sieved to achieve a particle size of less than 75µm. The washed sand (200 g) was then treated with a 1.0 M solution of H_2_SO_4_ (150 mL) in a mechanical stirrer at 600 rpm for a period of one hour; the resulting mixture was filtered and again washed with distilled water until a neutral pH was obtained and dried at 110°C for 12 h.

Iron was incorporated into silica using a sol–gel method. A Fe–Si–O_2_ sol–gel was produced by the hydrolysis of tetraethyl orthosilicate. The hydrolysis process started with a mixed solution consisting of 15 mL tetraethyl orthosilicate in ethanol (30 mL), distilled water (30 mL), and diluted hydrochloric acid (5 mL with a pH of approximately 2). The sol–gel mixture was magnetically stirred at 500 rpm for 3 h at room temperature to ensure complete hydrolysis and subsequent condensation. The hydrolyzed sol–gel was combined with 10 g of acid-activated (1M H_2_SO_4_ and washed 3 times with distilled water) beach sand that had undergone acid activation. The sand was added to the sol–gel mixture and stirred for an additional 60 min to ensure homogeneous distribution of iron-containing SiO_2_ species throughout the entire gel. The completed gel was aged at a temperature of 60 °C for a period of 18 h to obtain the produced Fe-SiO_2_.

Fly ash (300 g) was washed three times (1 L for each washing) with distilled water, dried at 110 °C, and then ground to a particle diameter of less than 75 µm using a sieve. The fly ash underwent acid treatment to expose the reactive aluminosilicate sites by preparing a suspension in 0.5 M HCl and heating it at a temperature of 60°C for 45 min while continuously stirring. After this treatment, the fly ash was filtered to remove excess acid, washed with distilled water until the pH of the filtration solution returned to neutral, and then dried at 110 °C for a period of 12 h.

**Fig. 3:**
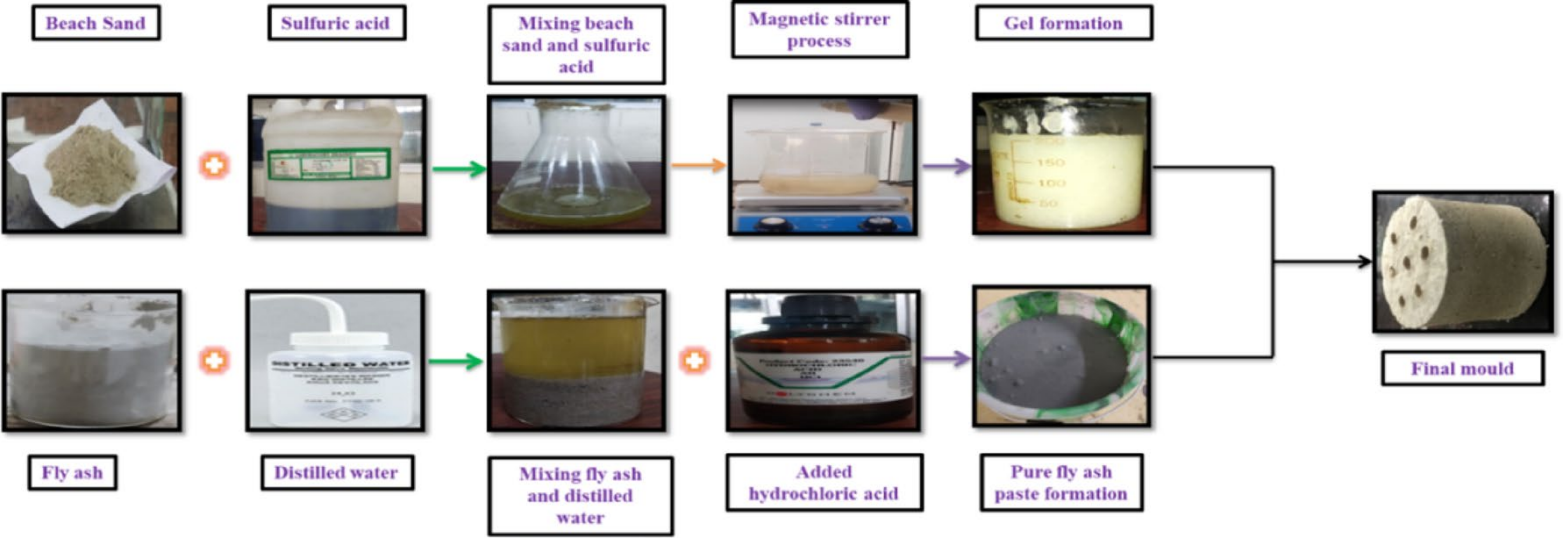
Synthesis of Fe_2_O_3_–SiO_2_/Al_2_O_3_catalyst material.

In order to make an equal-weight molecular mixture (hybrid precursor), a 1:1 ratio of Fe/SiO_2_ and the acid-treated fly ash were combined together. A plasticizer, which will equate to 7 wt% of the total solids was added to the mixture (in the form of 30 mL of deionized water) to enable the mixture to be soft and moldable. The resulting mixture was placed in a cylindrical PVC mold with a 50 mm diameter and a 50 mm height (with 6 longitudinal channels) and allowed to harden. The samples were dried at room temperature for 24 h and were subsequently thermally processed in a muffle furnace at 250 °C for 6 h using an increase in temperature of 3 degrees/minute. After cooling to room temperature, the rigid product of the combined Fe_2_O_3_/SiO_2_/Al_2_O_3_ material was removed from the PVC mold and placed into stainless steel housing for use as a SCR within a diesel engine exhaust emissions study. The principal components of the Fe_2_O_3_/SiO_2_/Al_2_O_3_ catalyst material as synthesis of the catalyst as procedure shown in Fig. 3 and its function as a catalyst are listed in Table 5.

**Table 5:** Physicochemical properties of test fuels determined using standard test methods^6^.

Raw material	Major components (wt%)	Minor/trace components (wt%)	Typical chemical formulae	Functional role in synthesis catalyst
Beach sand	SiO_2_ (85–95)	Al_2_O_3_ (1–3), Fe_2_O_4_ (1–3), TiO_2_ (1–2), ZrSiO_4_ (0.2–1). CaCO_3_ (< 1)	SiO_2_, FeTiO_2_, Fe_3_O_4_, ZrSiO_4_	Serves as silica source it provides high surface area and thermal stability and also enhances dispersion of Fe_2_O_3_ and Al_2_O_3_ phases
Potash	K_2_CO_3_ (85–90)	KCl (5–10), K_2_so_4_ (< 2)	K_2_CO_3_, KCl	Acts as binding agents as facilitate silica alumina interaction and promotes formation, improves catalyst porosity and thermal resistance

### Physicochemical and morphological studies of synthetic catalysts

FTIR spectroscopy was used for characterizing functional groups on the catalyst surface, determining the degree of metal–oxygen bond formation, and understanding the acidity of the catalyst during synthesis. FTIR spectroscopy was performed using a Nicolet iS50 spectrometer at ambient temperature over a 4000—400 cm^−1^ range. FTIR measurements were made of the fresh (before exhaust gas passes through the catalyst) and used (after exhaust gas passes through the catalyst) to determine their structural integrity and whether their chemistries changed during use in a diesel engine. FTIR spectroscopy measurements were made only as an ex-situ technique with no measurements taken under high temperatures. In NH_3_ adsorption experiments, samples were cleaned in a 500 ppm NH_3_/N_2_ gas mixture and spectra were recorded every 10 min until the point of saturation was reached for NH_3_ adsorption. The crystal structures of the catalyst molds were determined using Cu Kα radiation and powder XRD. The intensity data were acquired over Thomas’ 2 scanning angles from 5° to 80° at a scanning speed of 2°/min. Nitrogen adsorption and desorption isotherms were measured at − 195 °C using a micrometer analyzer. The specific surface area and pore size distribution were calculated using the BET method. SEM images were taken using a Zeiss EV050 to study the catalyst surface morphology, with scanning voltage and spot size changed to acquire high-resolution images at various magnifications.

### Experimental setup

**Fig. 4:**
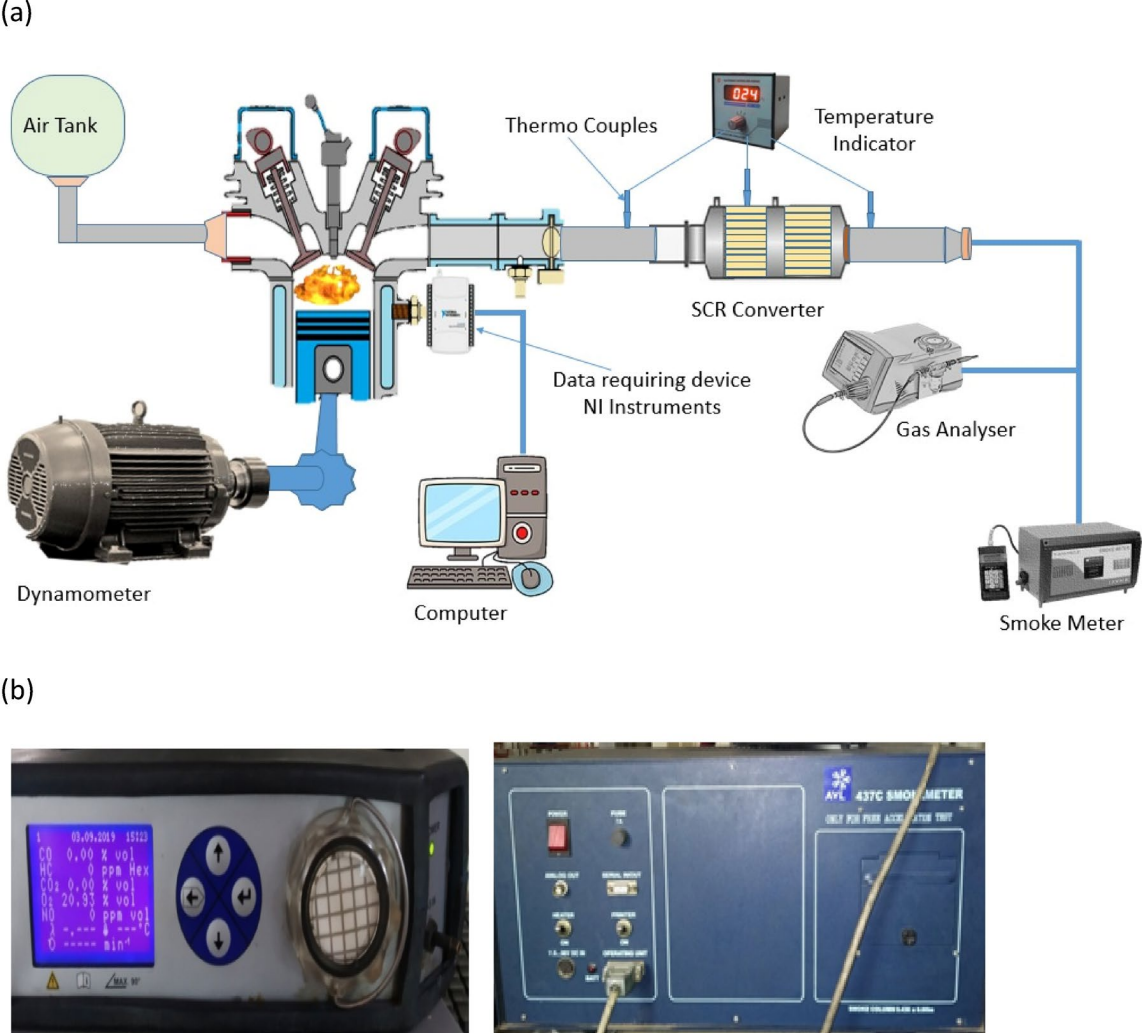
(**a**) Schematic engine setup. (**b**) Layout of engine and AVL Gas analyzer and Smoke meter.

The experiment was conducted using a 5.2 kW Kirloskar DI engine and an eddy current dynamometer. The engine had a four-speed, however little gearbox or forced induction. The exhaust gases analyzed in the tailpipe were assessed by an AVL gas analyzer. A smoke meter was also run in order to check smoke opacity. Also provided is a description of the engine layout and the AVL gas analyser and smoke meter (see Fig. 4a,b), and the engine specifications can be found in Table 6. The examination was conducted over four experimental trials to determine the effects of catalysts and fuel on exhaust gas emission analysis in the research engine.

- T1 trial of the experiment used diesel fuel.
- T2 trial used DPB fuel.
- T3 trial employed diesel fuel and SCR-based momold.4 trial utilized DPB fuel and an SCR-based mold.

**Table 6:** Engine specification.

S. No	Engine specifications	Engine details
1	Engine model	5.2kW diesel engine
2	Type of engine	Single cylinder
3	Number of Stroke	4
4	Cooling system	Air cooled
5	Operating speed	1500 rpm

To separate and evaluate the effect of the Fe_2_O_3_–SiO_2_/Al_2_O_3_ catalyst mold, emissions were measured under two conditions:

- Prior to the catalyst, the SCR was not placed inside the exhaust manifold (the lab called this the “baseline”).
- After the catalyst, the SCR mold was attached to the exhaust system.

The SCR was developed utilizing a mold to produce a removable mold configuration. The exhaust manifold was designed with an extra flanged area to accept the SCR in either configuration. In the “before-catalyst” condition, the SCR mold was removed and then observed more closely downstream of the exhaust gas exit. In the "after-catalyst" condition the molded section fit tightly into the flanged section of the manifold and did not provide an opportunity for leaking. Gas sampling probes were placed both before and after the SCR mold was added, so emissions differences could be exactly measured.

This approach guaranteed that the recorded reductions in emissions were solely the result of the impact of the catalyst and not because of any variations in the fuel.

The study tests included:

- T1: Engine fuelled with diesel,
- T2: Engine fuelled with DPB,
- T3: Engine Fueled with Diesel with a Molded SCR Catalyst.
- T4: Engine Fueled with DPB with a Molded SCR Catalyst.

We examined exhaust gasses and smoke opacity with exhaust gas analysers type AVL DIGAS 444 to measure the exhaust emissions at nominally: NO, CO, HC, CO_2,_ and smoke opacity. Ensure repeatability, the ambient conditions were maintained at 30 °C ± 2 °C and 60–70% relative humidity.

The standard equation for Brake Thermal Efficiency (BTE) is:

$${\mathrm{BTE}}\, = \,\left( {{\mathrm{BP}}/{\dot{\mathrm{m}}\mathrm{f}}\, \times \,{\mathrm{CV}}} \right)\, \times \,{1}00,$$

Where BP = brake power (kW), ṁf = fuel mass flow (kg/s) and CV = calorific value of the fuel (for diesel, CV = 44,460 kJ/kg, for DPB CV = 43,555 kJ/kg). The Fe_2_O_3_–SiO_2_/Al_2_O_3_ catalyst was fabricated into a monolithic honeycomb structure suitable for mounting in the exhaust pipe of the 5.2 kW single-cylinder diesel engines. The cylindrical monolith had a length of 5 cm and a diameter matching the exhaust pipe diameter (3.5 cm). The honeycomb consisted of square channels (≈ 2 mm × 2 mm) with a thickness of approximately 0.5 mm, corresponding to a channel density of about 400 CPSI, providing adequate geometric surface area while maintaining low flow resistance. Experimental measurements under real exhaust conditions confirmed that the pressure drop across the monolith remained below 1.5 kPa over the operating temperature range of 150–600 °C, which is within acceptable back pressure limits for the engine and did not adversely affect BTE or fuel consumption. These results confirm that the catalyst’s honeycomb geometry is suitable for engine-scale SCR application without impeding exhaust flow. The mass flow rate of the fuel was calculated by timing how long it took to combust a known amount of fuel. Then the engine specification and SCR catalyst are operating as shown in Tables 6 and 7.

**Table 7:** SCR catalyst operating and testing conditions.

Parameter	Value/Range	Symbol	Unit	Remarks
GHSV	35,000	GHSV	h^−1^	Calculated from exhaust gas flow rate and catalyst volume
Exhaust gas temperature	150–600	T	⁰C	Measured using thermocouple upstream and downstream of SCR
Urea Injection Rate	0.5–0.55	–	gs^-1^	Adjusted based on total NO concentration and engine load
Catalyst Volume	0.15	V (cat)	L	Geometric volume of honeycomb monolith
Exhaust Flow Rate	6	Q	m^3^h^−1^	Estimated form engine displacement and speed
NH_3_: NOx molar ratio	–	α	–	Controlled indirectly via urea dosing; not directly measured
NO/NO_2_ ratio	–	–	–	Not measured due to instrumentation limitation
Exhaust Oxygen Concentration	–	O_2_	% vol	Not measured; lean diesel operation assumed

## Results and discussion

### L–H mechanism of synthesis catalytic mold

The NH_3_—SCR reaction over the Fe_2_O_3_–SiO_2_/Al_2_O_3_ catalyst proceeds through a surface-mediated pathway that is consistent with a Langmuir–Hinshelwood-type mechanism, in which both NO and NH_3_ interact with the catalyst surface prior to reaction. NO is preferentially adsorbed on redox-active Fe^3+^/Fe^2+^ sites, while NH_3_ is anchored on Lewis and Bronsted acid sites associated mainly with the Al_2_O_3_ support. Surface-adsorbed NH3 undergoes activation to form reactive NH2 and NH4+ species, which subsequently react with NO-derived surface species to produce N2 and H2O.

**Fig. 5:**
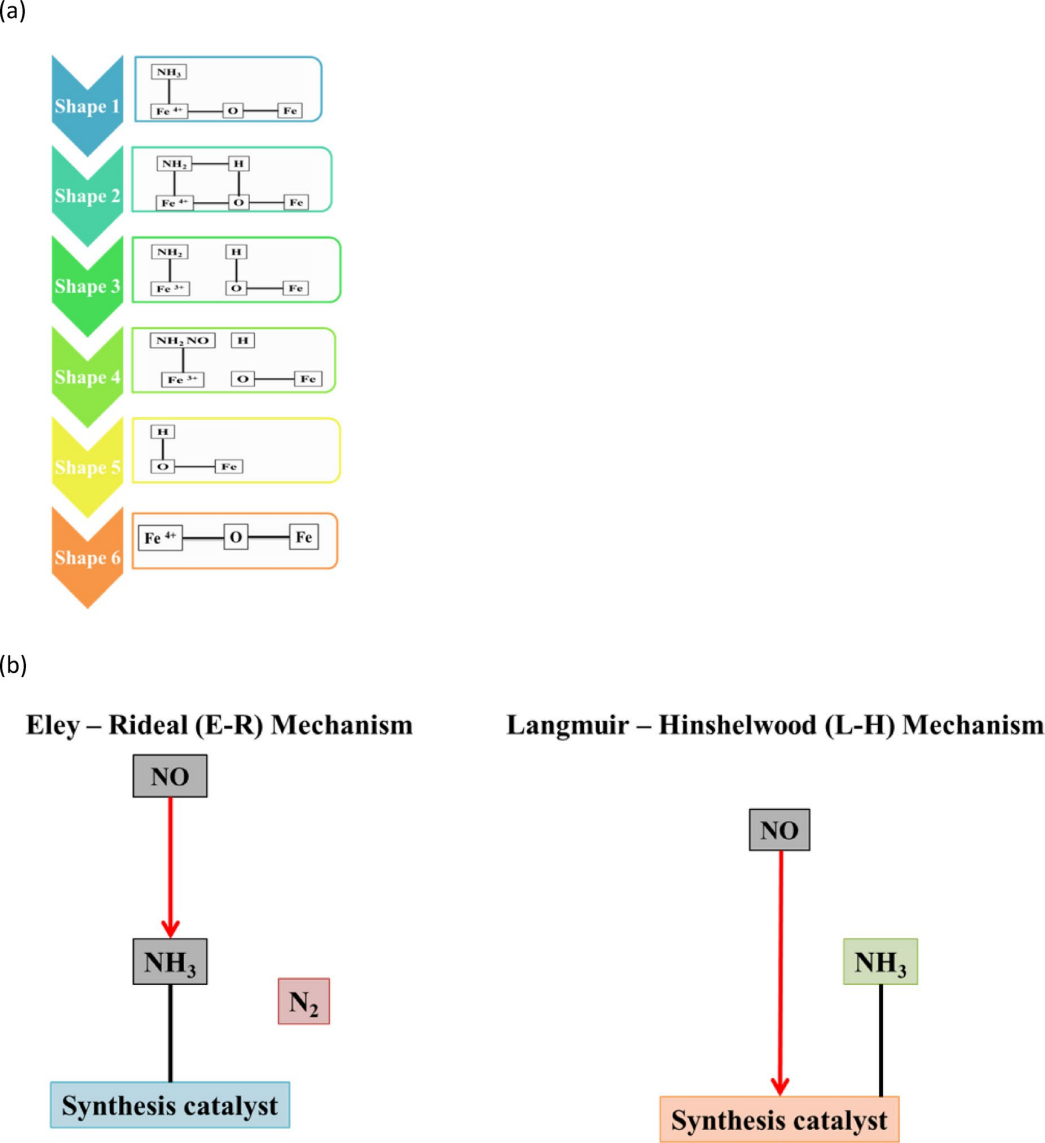
(**a**) L–H Mechanism reacts to the synthesized catalyst. (**b**) L–H Mechanism reacts to the synthesized catalyst.

The high SCR activity of the Fe_2_O_3_–SiO_2_/Al_2_O_3_ system is attributed to the synergistic coupling of redox and acidic functionalities. SiO_2_ promotes uniform dispersion of iron oxide and inhibits thermal sintering, while Al_2_O_3_ provides stable acid sites required for effective NH_3_ adsorption. Interfacial Fe–O–Si and Fe–O–Al interactions facilitate Fe^3^⁺/Fe^2^⁺ redox cycling and NO activation. In-situ FTIR observations of coordinated NH_3_ species and nitrate/nitrite intermediates indicate the coexistence of surface-adsorbed reactants and are consistent with a surface-controlled SCR process. The reaction scheme illustrated in Fig. 5 (a) and (b) is therefore presented as a conceptual description based on spectroscopic observations and catalytic performance data. Further kinetic and isotopic studies will be undertaken to provide additional mechanistic clarification.

### Adsorption–desorption mechanism of synthesis catalyst

**Fig. 6:**
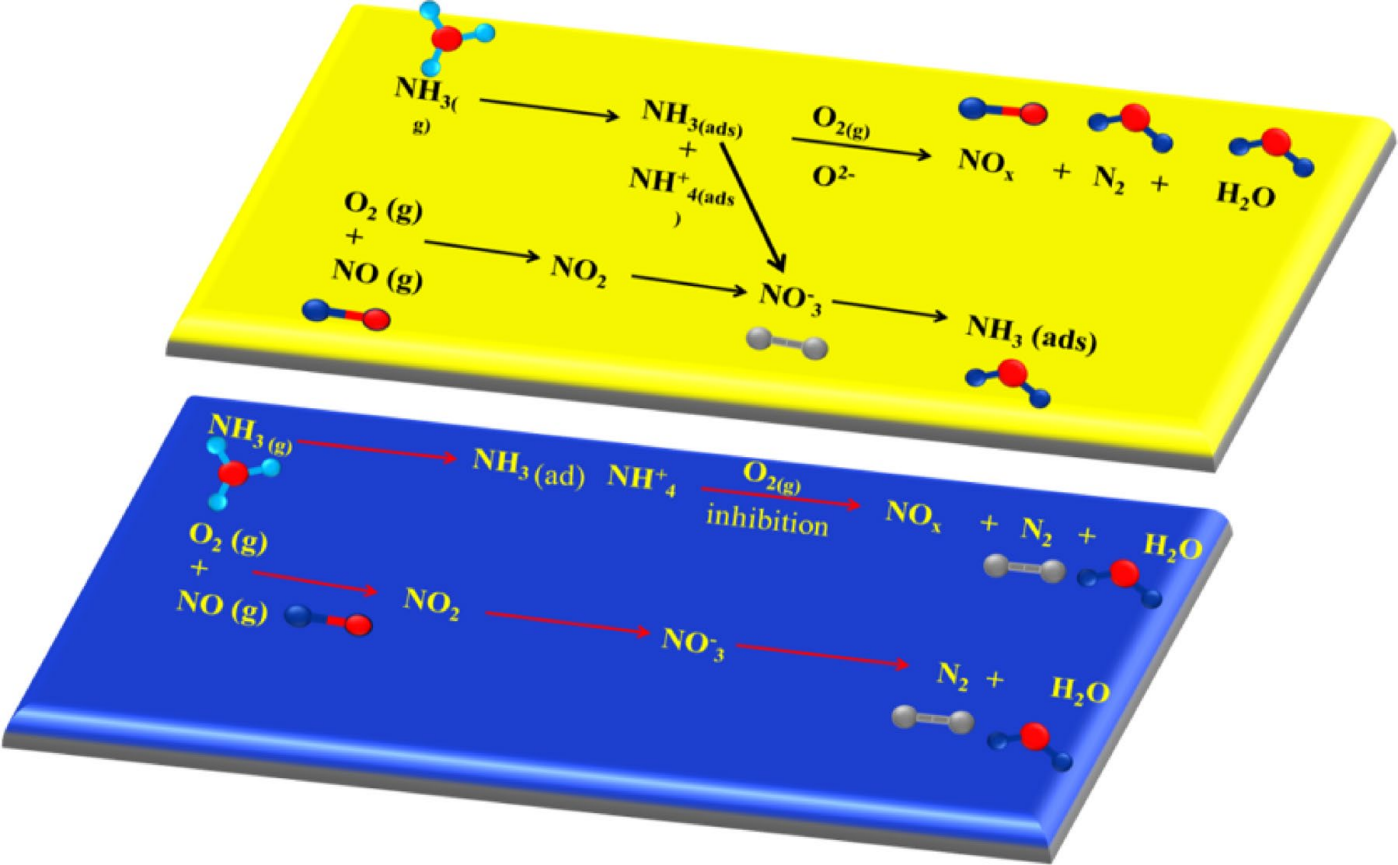
Mechanism of synthesis catalyst Fe_2_O_3_–SiO_2_/Al_2_O_3_.

During the period leading up to 150 °C, all things being equal, the low efficiency of the mold catalyst was first evident because the exhaust gases flowed away from the catalyst. As the temperature of the exhaust gases increased step by step until reaching 600°C, increased catalytic performance was observed, indicating much better performance at higher temperatures. The physical behavior of the exhaust gases passing through the mold catalytic is depicted in Fig. 6. Both high and low temperature operation conditions demonstrate effective catalytic performance by means of a molded catalyst. In addition, the results of other tests such as XRD, FTIR and SEM demonstrate that the Catalytic mold Fe_2_O_3_–SiO_2_/Al_2_O_3_was able to function well below the thermal limits, yet the other catalysts show a lower efficiency as the temperature rises. Only at the highest temperatures does it exhibit significantly reduced quantities of NO emissions at the mold catalyst surface and therefore it is expected that the efficacy will continue to show this range of effectiveness.

### FTIR study

A FTIR was employed to investigate the surface adsorption behavior and reaction intermediates formed on the Fe_2_O_3_—SiO_2_/Al_2_O_3_ catalyst during the NH_3_-SCR process. The FTIR would identify molecular vibrations and surface functional groups that experience altered chemical bonding when gas and solids come in contact. This technique provides spectroscopic insight into surface species and reaction intermediates associated with NO reduction. In the present system, urea served as the precursor for NH_3_ generation, decomposing in the presence of water vapor and exhaust heat according to:

$${\text{CO }}\left( {{\mathrm{NH}}_{{2}} } \right)_{{2}} + {\text{ H}}_{{2}} {\text{O }} \to {\text{ 2NH}}_{{3}} + {\text{ CO}}_{{2}}$$

The produced NH_3_ then underwent a reaction with the NO that was already present in the exhaust stream over the Fe_2_O_3_—SiO_2_/Al_2_O_3_catalyst surface. The generated NH_3_ adsorbed on both Lewis and Bronsted acid sites associated with Fe-containing surface environments and oxygen-deficient regions of the catalyst structure, respectively. Finally, in the formation of coordinated and ionic ammonium species were key intermediates in the SCR mechanism. In the FTIR spectra, characteristic absorptions were detected at approximately 1892 cm^-1^ and 487.5 cm^-1^, which are commonly assigned to coordinated NH_3_ species interacting with Fe-associated Lewis’s acid sites. After the introduction of mixed NO and O_2_ gases, as expected, these regions of NH_3_ absorption quickly weakened, indicating the consumption of adsorbed NH_3_ during surface reactions with NO. Some of the NH_3_ residing on Lewis’s acid sites, on the other hand, was stable; suggesting that NH_3_ bound to these sites may contribute to sustained SCR activity.

The appearance of a new band about 495 cm^−1^ during the NO–NH_3_ interaction indicates the formation of bridging nitrate species associated with NO adsorption on Fe-containing surface sites. Also, negative bands appearing at about 625 and 719 cm^−1^ are likely due to sulfate groups being displaced from the surface due to NO induced substitution This suggests that NO interaction involves lattice-oxygen participation under the studied SCR conditions. These spectroscopic features are consistent with redox participation involving Fe^3^⁺/Fe^2^⁺ species, as commonly reported for Fe-based SCR catalysts, although the exact oxidation-state distribution cannot be directly quantified in the absence of XPS analysis. The presence of coordinated ammonium at Lewis acid sites and ionic ammonium on Bronsted acid sites indicates that the surface acidity is balanced, which is considered favorable for the conventional NH_3_-SCR reaction pathway.

**Fig. 7:**
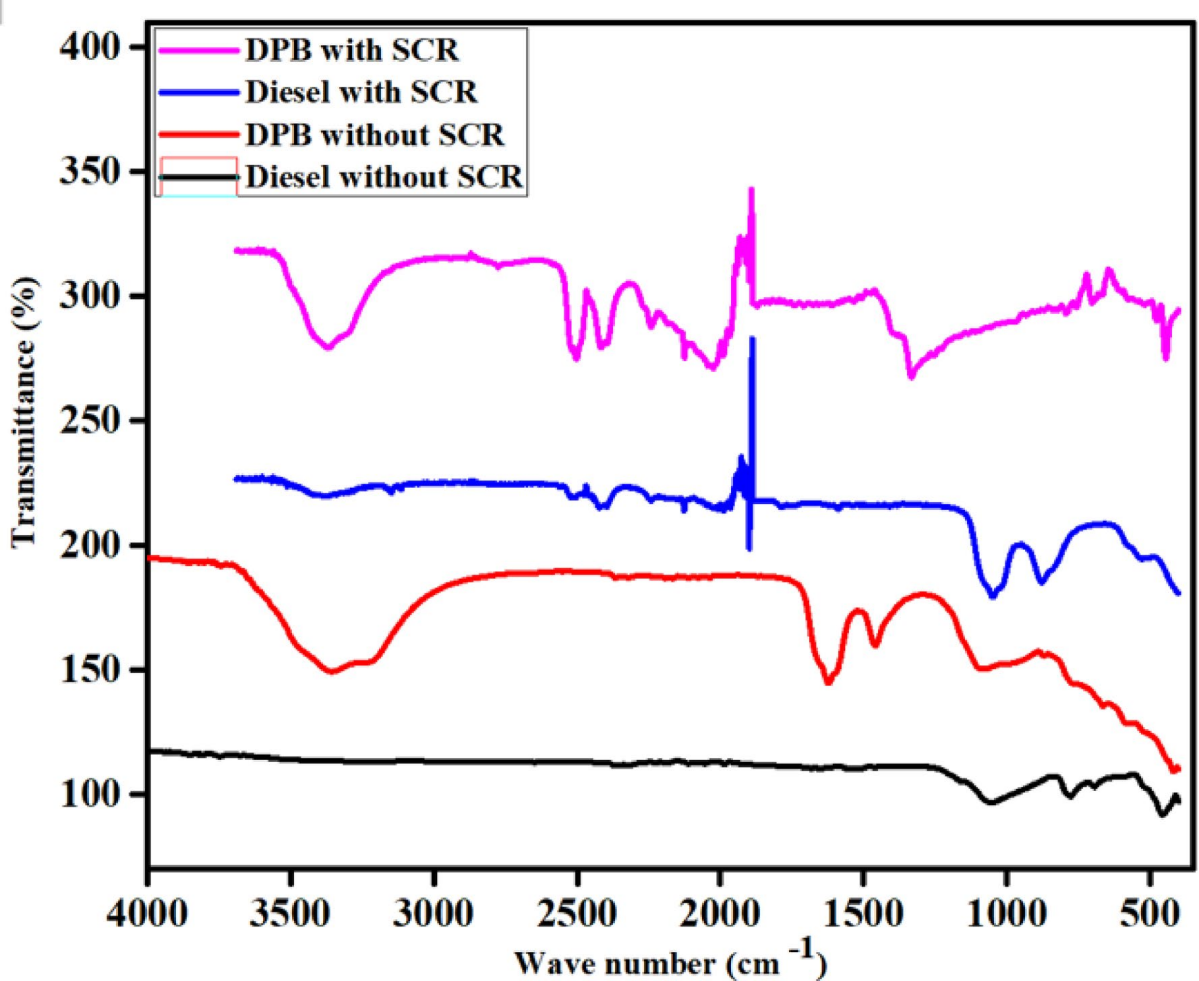
FTIR study for Synthesis catalyst Fe_2_O_3_–SiO_2_/Al_2_O_3_.

In summary, FTIR analysis indicates that the Fe_2_O_3_–SiO_2_/Al_2_O_3_catalyst mold possesses a stable distribution of acidic sites and Fe associated redox-active surface environments capable of sustaining NH_3_ adsorption and nitrate formation during SCR operation capable of retaining NH_3_ for a long time-period, assisting in the formation of nitrates and promoting electron transport. The amalgamation of these site properties yields a highly active, stable and reproducible process for the NO conversion during the SCR process. The entire process of urea hydrolysis to generate NH_3_, the NH_3_ adsorption, NO reduction, highlights the catalytic activity and operational stability of the Fe_2_O_3_–SiO_2_/Al_2_O_3_ system for de-NO_x_ applications under diesel engine exhaust conditions (Fig. 7).

### XRD study

The XRD analysis of the Fe_2_O_3_–SiO_2_/Al_2_O_3_ catalyst reveals a predominantly amorphous structure, with a broad SiO_2_ background and weak, broadened peaks attributable to poorly crystalline γ-Al_2_O_3_. The absence of distinct diffraction peaks corresponding to crystalline iron oxide phases (e.g., α-/γ-Fe_2_O_3_ or Fe_3_O_4_) strongly suggests that iron does not exist as large, well-ordered crystallites. Instead, iron is likely present in a highly dispersed, X-ray amorphous state, possibly as nanoscale clusters, surface-anchored species, or within an amorphous mixed-oxide matrix stabilized by Fe–O–Si and Fe–O–Al interactions.

**Fig. 8:**
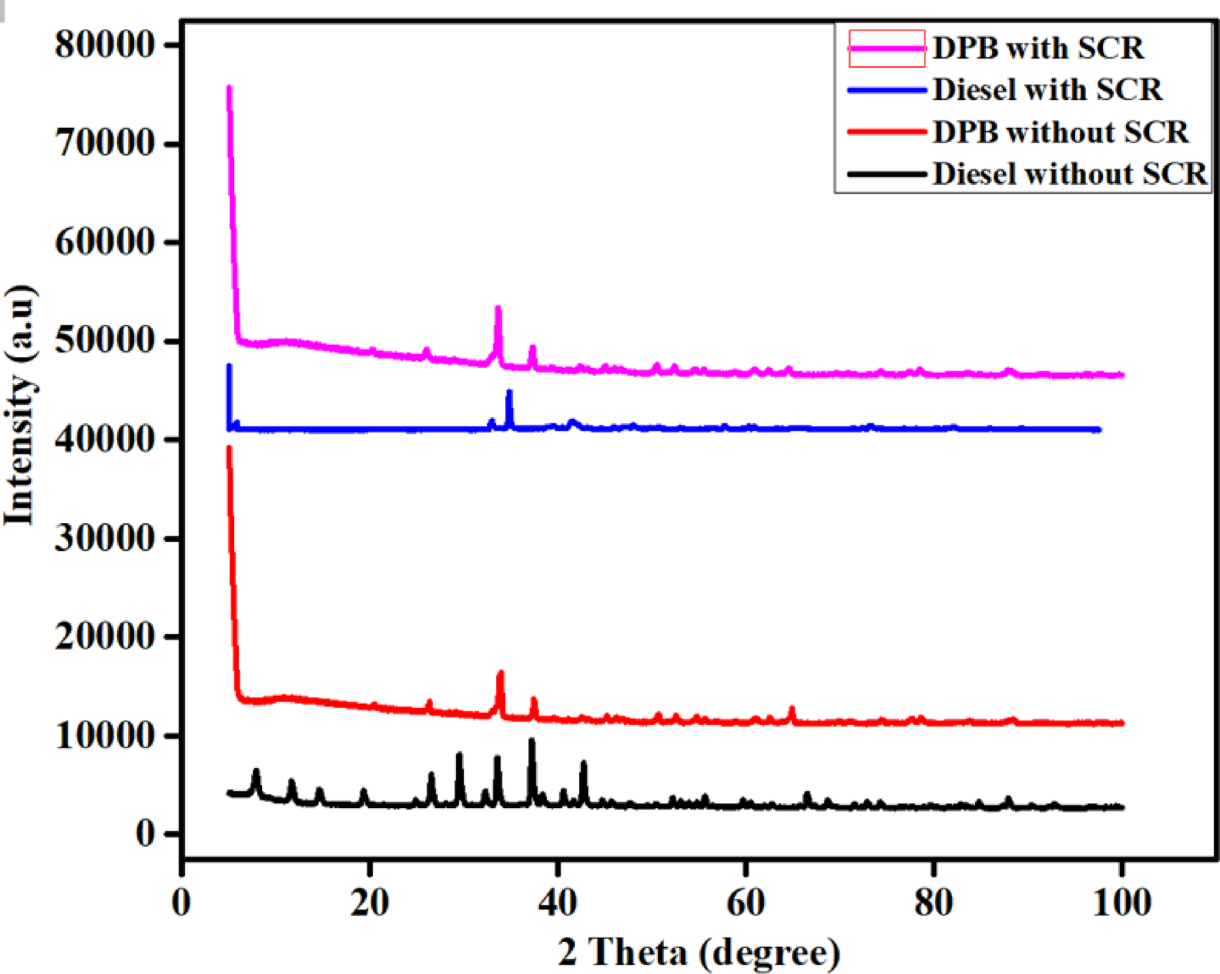
XRD Analysis of Fe_2_O_3_–SiO_2_/Al_2_O_3_Catalyst in Diesel and DPB Systems: Comparison with and without Catalyst Incorporation.

The XRD indicates a highly dispersed, non-crystalline Fe phase rather than well-defined Fe_2_O_3_ crystallites. FTIR data confirms the presence of surface-active Fe species capable of NH_3_ adsorption and nitrate formation, consistent with catalytic activity despite XRD invisibility. The spectroscopic evidence supports the functional role of Fe in the SCR mechanism, even in the absence of long-range crystalline order as shown in Fig. 8.

### SEM analysis

As described in Table 8, an SEM morphological examination of the synthesized catalyst indicates that the structure of the Fe_2_O_3_–SiO_2_/Al_2_O_3_ catalyst remained unchanged before and after reactions took place with flowing exhaust gas. This indicates that the particles were in different orientations and were of different sizes. The catalyst coating was smooth, presenting small-sized pores and surface fractures uniformly distributed throughout the surface. This suggests that the structural integrity remained stable throughout the reactions and aligned with existing literature regarding the impact of thermal and chemical stressors on supported transition metal oxide systems^35^.

**Table 8:**
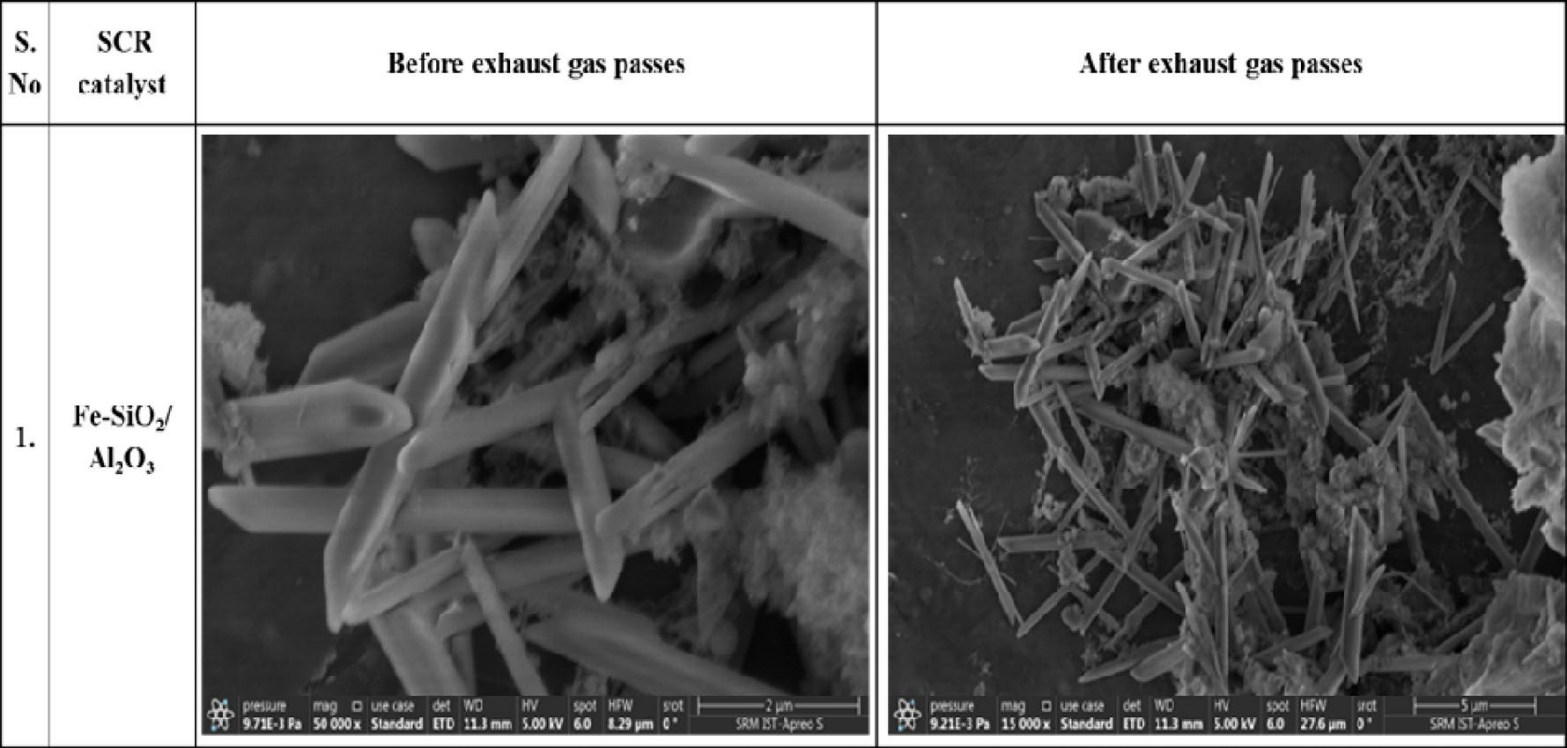
SEM analysis of synthesis catalyst.

### BET study

**Fig. 9:**
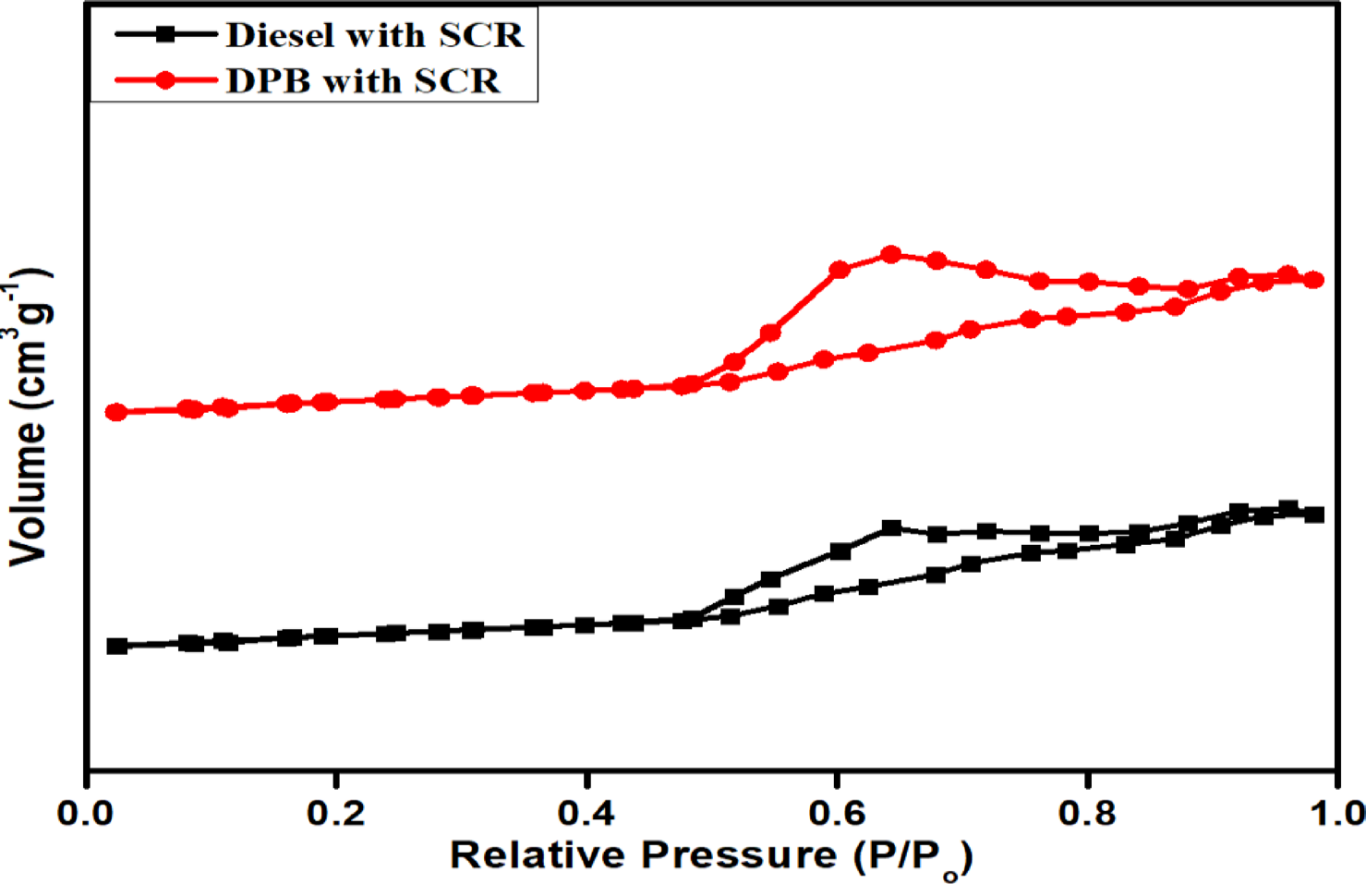
BET Analysis of Fe_2_O_3_–SiO_2_/Al_2_O_3_Catalyst in Diesel and DPB Systems.

The BET method was used to determine the porosity, pore volume/diameter, and surface area of each synthesized catalyst (Fig. 9). The shapes of the adsorption isotherms were Type IV indicating that the catalyst has a mesoporous structure, and this characteristic remained after the catalysts were used in diesel and DPB exhaust applications. When Fe_2_O_3_ was added to the SiO_2_/Al_2_O_3_ supports, the BET surface area decreased progressively with the total pore volume, and the average pore diameter decreased from 12.8 to 9.8 nm. This reduction in pore diameter can be attributed to the partial filling and blocking of the pores caused by the dispersed iron oxide particles, which is a common occurrence with Fe-loaded oxide catalysts.

Although BET measurement indicated a decrease in surface area, the Fe_2_O_3_–SiO_2_/Al_2_O_3_ catalyst still had the highest catalytic activity. This apparent contradiction can be explained by understanding that the quality and accessibility of an active site contribute more to the overall performance than the total surface area.

The FTIR data showed that the Lewis acid site and Bronsted acid site were very accessible, while the presence of well-dispersed Fe^3^⁺/Fe^2^⁺ redox centres allowed good ammonia adsorption and efficient NO reduction. Using Fe_2_O_3_ to make SCR supported on Al_2_O_3_-SiO_2_ resulted in a decrease in total amount of pore volume (i.e. BET surface area), but the SCR performance of Fe_2_O_3_ over Al_2_O_3_-SiO_2_ was improved due to the introduction of isolated (monomeric) and oligomer iron species (Fe^3+^) into the Al_2_O_3_–SiO_2_ framework as active sites for NH_3_ adsorption and NO reduction. Therefore, although the BET surface area has decreased, the presence of mesopores allows for effective diffusion of exhaust gasses into the catalyst and therefore minimizes the effect of internal mass transport limitations under typical operating conditions for diesel engines. Additionally, the presence of Fe_2_O_3_ creates a source for the formation of Lewis acid sites (i.e., Fe^3+^/Fe^2+^ redox pairs) which stimulates SCR reactions. Many studies have observed that Fe_2_O_3_–SiO_2_ performance is enhanced because of its unique active site chemistry despite having lower BET surface areas^42^. In addition, the mesopores structure of Fe_2_O_3_-SiO_2_ has pored diameters that are < 10 nm and therefore will also enhance the mass transfer of exhaust gasses into the catalyst and reduce the effect of internal mass transport limitations under typical operating conditions for diesel engines (high space velocity)^43^. These results indicate that while the total amount of BET surface area was reduced, the presence of mesopores increases the amount of area available for active site access.

#### Catalyst correlation between BET, FTIR and conversion efficiency of exhaust emission

Research conducted with BET and FTIR suggests that the catalytic behavior of Fe_2_O_3_–SiO_2_/Al_2_O_3_ for altering NO, HC and CO are highly dependent upon the physicochemical properties of the surface. The BET surface areas indicated that the addition of Fe led to lower values for both specific surface areas and mean pore diameter. However, the catalyst showed an improvement in conversion efficiency, which suggests that it is the type and availability of active sites that relate more closely to catalytic behavior than does surface area alone. Furthermore, the minor narrowing of the mesopore may enable improved diffusion and contact between the exhaust emission gas molecules and the active catalytic domains, resulting in more efficient oxidation and reduction reactions under dynamic engine operating conditions.

FTIR spectroscopy further clarified the mechanistic facets relevant to catalyst functioning. It was evident that coordinated NH_3_ species exist on Lewis and Bronsted acid sites tied to SCR reactions; thereafter, the rapid disappearance of these bands upon addition of NO and O_2_ indicated that verses of deposited NH_3_ played an active role in NO reduction mechanisms. The significance of NH_3_ on Lewis acid sites demonstrates that the catalyst structure tolerated oxidative environments and still performed functions as an SCR. The clear bands of bridging nitrates and sulfate displacement also collectively support the prospect of active surface chemistry assisting in the subsequent NO conversions. Conclusively, the results from BET and the FTIR mean a complete context that might explain why the conversion efficiencies remained high. BET gave complete coverage of good pore structure and the dispersion of active metal species. Whereas FTIR offered a testament to reactant surface chemical reactivity that may reduce NO, HC and CO, singularly or together. The apparently established structural-surface chemistry connection appears that Fe_2_O_3_–SiO_2_/Al_2_O_3_ may function as a better catalyst under catalytic thermal-heat load conditions when lean-burn diesel.

### BTE performance

**Fig. 10:**
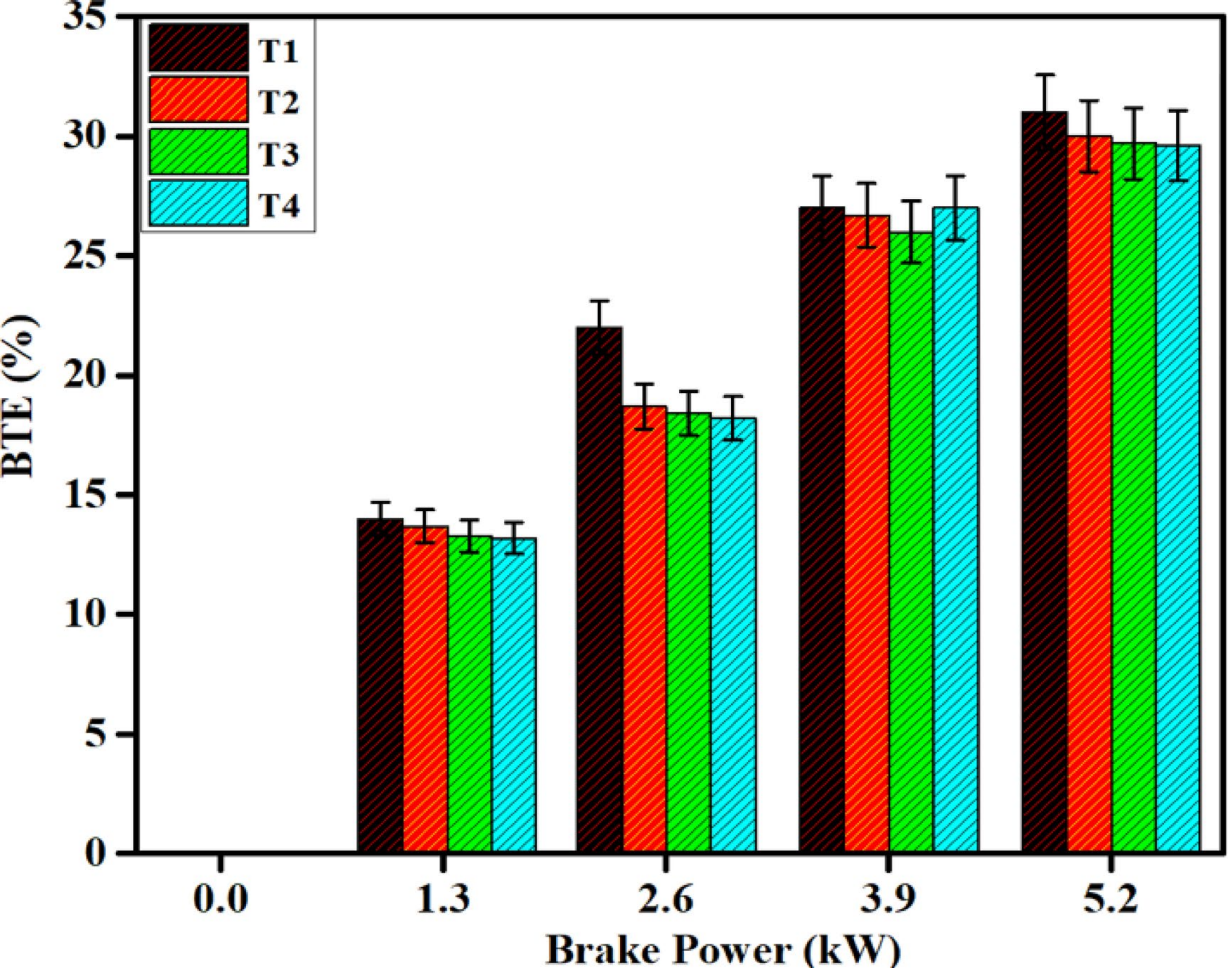
Difference in BTE with brake power for both diesel and DPB with and without a catalyst.

The relationship between BTE and brake power is illustrated in Fig. 10, comparing the load at the lowest and highest. DPB with SCR affects BTE slightly higher when the load is at its highest in comparison to diesel without SCR. This happens because DPB has lower calorific value and higher viscosity to affect the flow of the fuel. Slightly lower BTE with SCR plus DPB over diesel fuel was observed, which was a reduction of 2.86%.

### Specific fuel consumption (SFC)

**Fig. 11:**
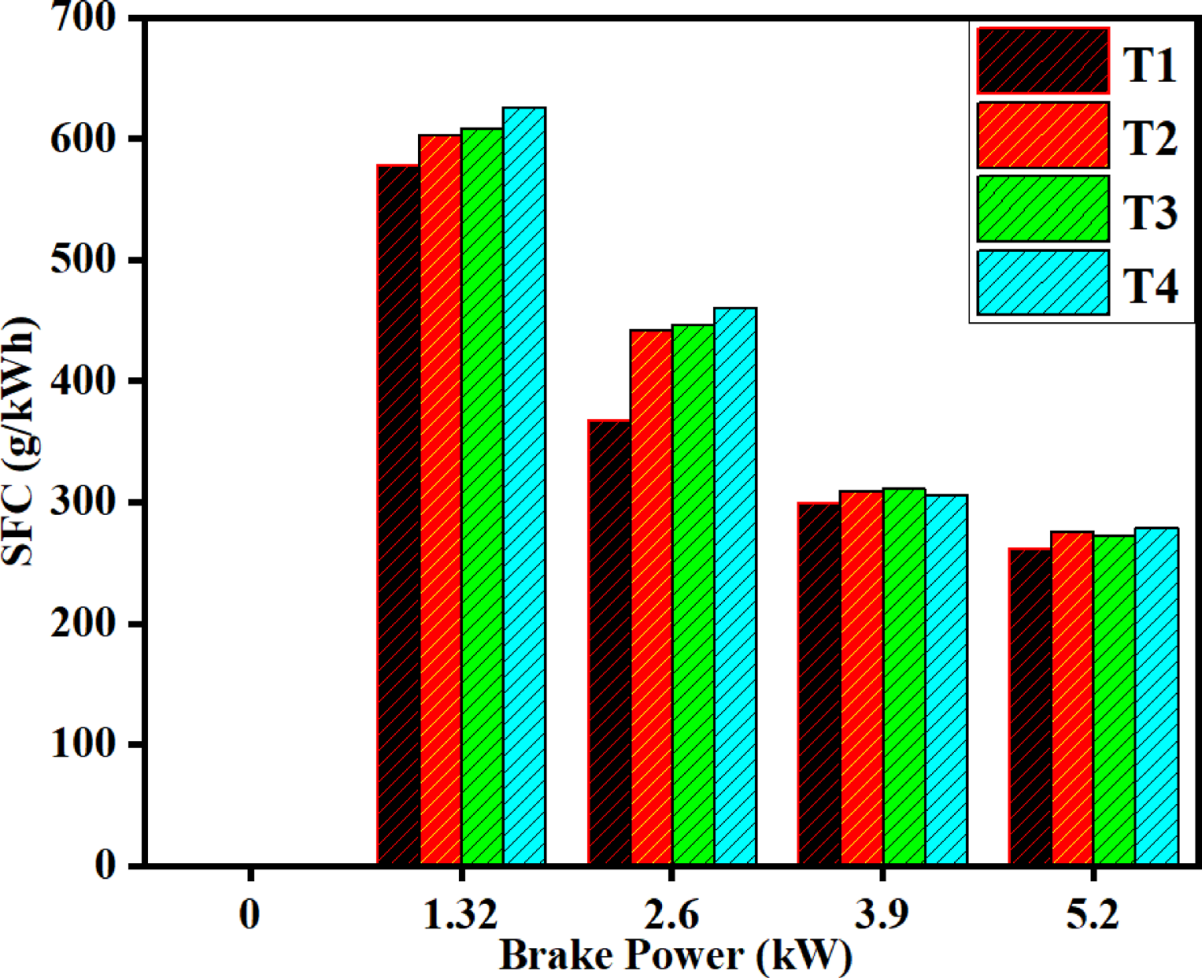
Difference in SFC with brake power for both diesel and DPB with and without a catalyst.

As demonstrated in Fig. 11, SFC decreases with increasing brake power across all fuels. This translates into greater combustion efficiency with higher load. The DPB blend has SFC values that are slightly higher than diesel consistently, due to DPB’s lower calorific value along with lower BTE. The addition of the SCR system increases SFC slightly due to the little increase in exhaust back pressure. The maximum load SFC values are 261.2 g/kWh for diesel and 275.5 g/kWh for DPB, which results in a 5.4% increase for the blend. The figure demonstrates how SFC evolves with load for diesel, DPB and SCR-assisted configurations for the two fuels. For all fuels, SFC drops with an increase in engine load. Higher engine loads mean a more complete combustion with lower relative heat loss. DPB requires slightly higher SFC values when compared to diesel under the load conditions investigated. This is due to DPB’s lower calorific value (43,555 kJ/kg), along with a lower BTE (2.86% lower than diesel).

### Exhaust emission

#### NO emission

In combustion processes characterized by elevated temperatures, the oxidation of nitrogen occurs swiftly and leads to NO emissions. The generation of NO, as described by the combustion theory, occurs from three principal reactions: thermal, prompt, and fuel mechanism^29,30^. The thermal mechanism is the principal way NO is produced in a diesel engine. NO production increases as temperature rises due to an increase in combustion efficiency in the cylinder.

There are emissions of NO for both diesel and DPB fuels, but there are great differences. For DPB, the NO emissions at the exhaust were slightly greater due to the characteristic that the ignition delay is longer, which increased the combustion of the premixed phase and produced an increase in heat release rate. This increases the temperature inside the cylinder would also favor NO production; therefore, both emissions of NO from DPB were greater than those of diesel.

**Fig. 12:**
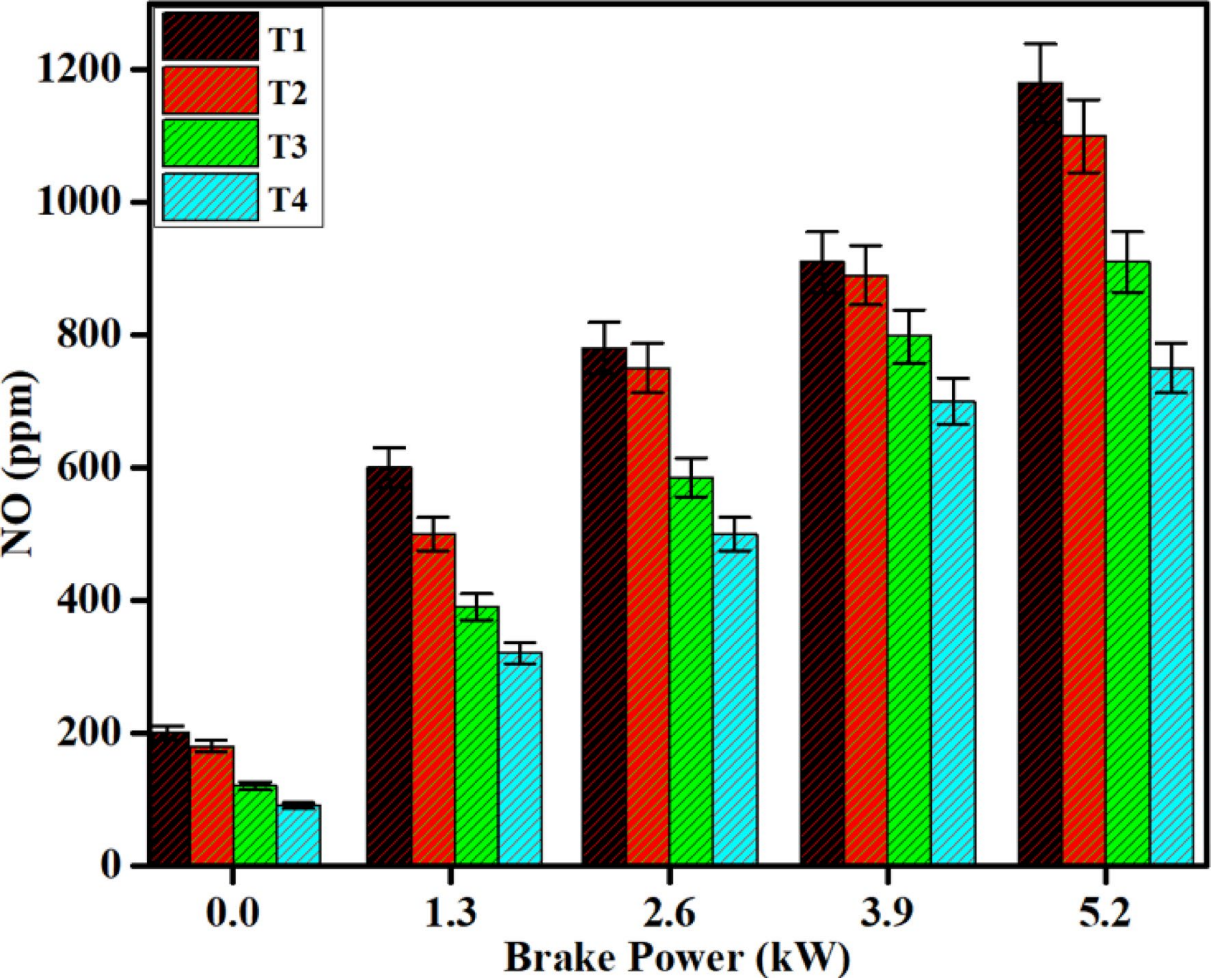
Difference in NO emissions with brake power for both diesel and DPB with and without a catalyst.

Despite SCR systems, it is still difficult to decrease NO emissions from diesel and DPB combustion. However, it can have a significant impact on reducing NO emissions when using a Fe_2_O_3_–SiO_2_/Al_2_O_3_ catalyst support mold in the urea-SCR system. This study evaluated the effects of the Fe_2_O_3_–SiO_2_/Al_2_O_3_ catalyst support mold on NO emissions from a diesel engine fueled with diesel and DPB fuels. The results showed that under varying load conditions NO emissions were reduced by approximately 68% and 75% using the SCR catalyst for diesel and DPB fuels, respectively (Fig. 12).

The SCR synthesis catalyst supporting structure lowers NO emissions from the dual fuel burner under various load conditions, compared to diesel and DPB fuel, with or without catalyst. Specifically, the SCR lowers NO emissions from both fuel types (in a dual burner configuration) by 80% for diesel and 85% for DPB when at peak loads. Resitgolu et al.^48^ indicated that the Al_2_O_3_–Nb_2_O_5_/CeO_2_/Fe_2_O_3_(ANCF) reduces NO emissions by 80.29%. The NO conversion for ANCF, the catalyst form, is only 53.45%, a direct comparison with the Fe_2_O_3_–SiO_2_/Al_2_O_3_ synthesis catalytic support. Although single-stage Fe-based SCR systems typically exhibit moderate NO conversion, the comparatively high reduction observed in the present study is attributed to the dual SiO_2_–Al_2_O_3_ support, which improves iron dispersion, surface acidity, and redox stability, as confirmed by BET and FTIR analyzes. Therefore, the maximum NO reduction of approximately 85% observed at full load is directly supported by raw exhaust concentration data and reflects favorable steady-state engine conditions rather than unrealistically idealized performance. Then the comparison of NO concentration with and without SCR reduction was shown in numerical Table 9.

**Table 9:** Raw NO concentrations and conversion efficiency for Fe_2_O_3_–SiO_2_/Al_2_O_3_SCR catalyst.

Engine load (%)	Exhaust gas temperature (°C)	NO concentration before SCR (ppm)	NO concentration after SCR (ppm)	NO reduction (%)
25	210–230	410	180	56.1
50	270–300	620	190	69.4
75	330–370	820	165	79.9
100	390–450	980	145	85.2

#### HC emission

**Fig. 13:**
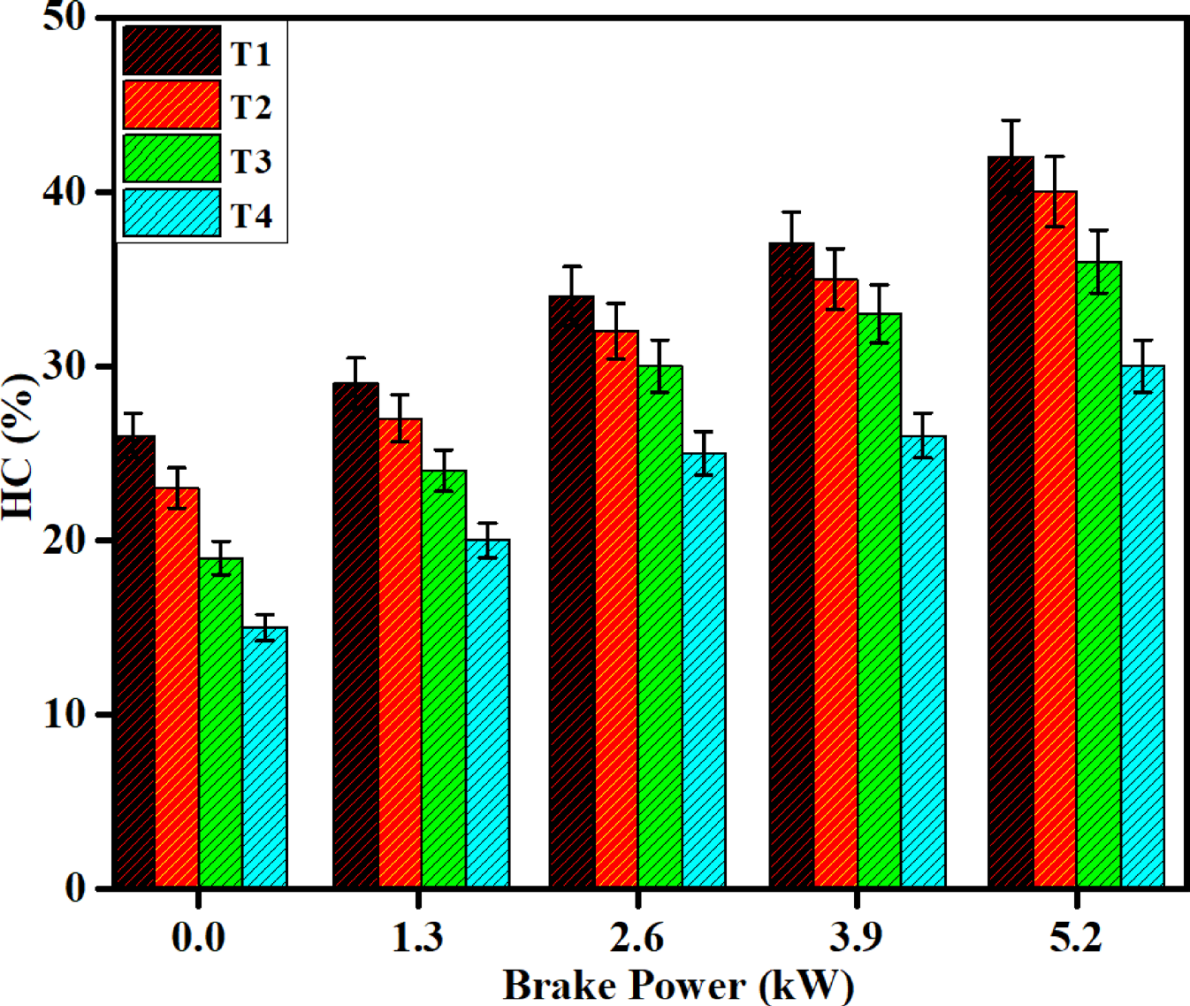
Difference in HC emissions with brake power for both diesel and DPB with and without a catalyst.

HC emissions were compared to break power for diesel and DPB fuels both with and without SCR catalytic systems. The primary contributors to the production of HC emissions are as follows, over-lean zones, poor mixing and flame quenching at the wall. HC emissions consist of mostly unburned portions of the fuel. Both diesel and DPB fuels contain a lot of aromatic compounds which tend to increase HC emissions because the aromatic compounds take longer to ignite and tend to burn for less time than non-aromatic. Therefore, diesel fuel emitted more HC emissions than DPB fuel. As shown on Fig. 13, HC emissions were reduced by 55% for diesel with SCR catalyst and 65% for DPB fuel with SCR catalyst.

Compared to the results reported by Resitgolu et al., in their comparative analysis, it was found that an ANCF catalyst reduced HC emissions by 52%. The Fe_2_O_3_–SiO_2_/Al_2_O_3_ synthesis catalyst, however, has several clear advantages over ANCF for the storage and release of oxygen. The Fe_2_O_3_–SiO_2_/Al_2_O_3_ catalyst has a far better HC conversion, attaining 53.45%, versus an HC conversion of 52% for ANFC. This improved HC conversion was due to the catalyst capability to maintain an oxidizing atmosphere throughout the entire period of HC oxidation to achieve further commitment of the HCs to full oxidation. SiO_2_ also improves the redox characteristics of the catalyst, which will improve HC oxidation^40^. In addition to the Fe_2_O_3_–SiO_2_/Al_2_O_3_ combination aiding in the stability of the catalyst at elevated temperatures, the catalyst should maintain improved fuel conversion over the temperature range wider than ANCF. It is possible that Fe_2_O_3_–SiO_2_/Al_2_O_3_ option opens further possibilities for enhanced HC conversion and could be a more efficient catalyst option.

#### CO emission

**Fig. 14:**
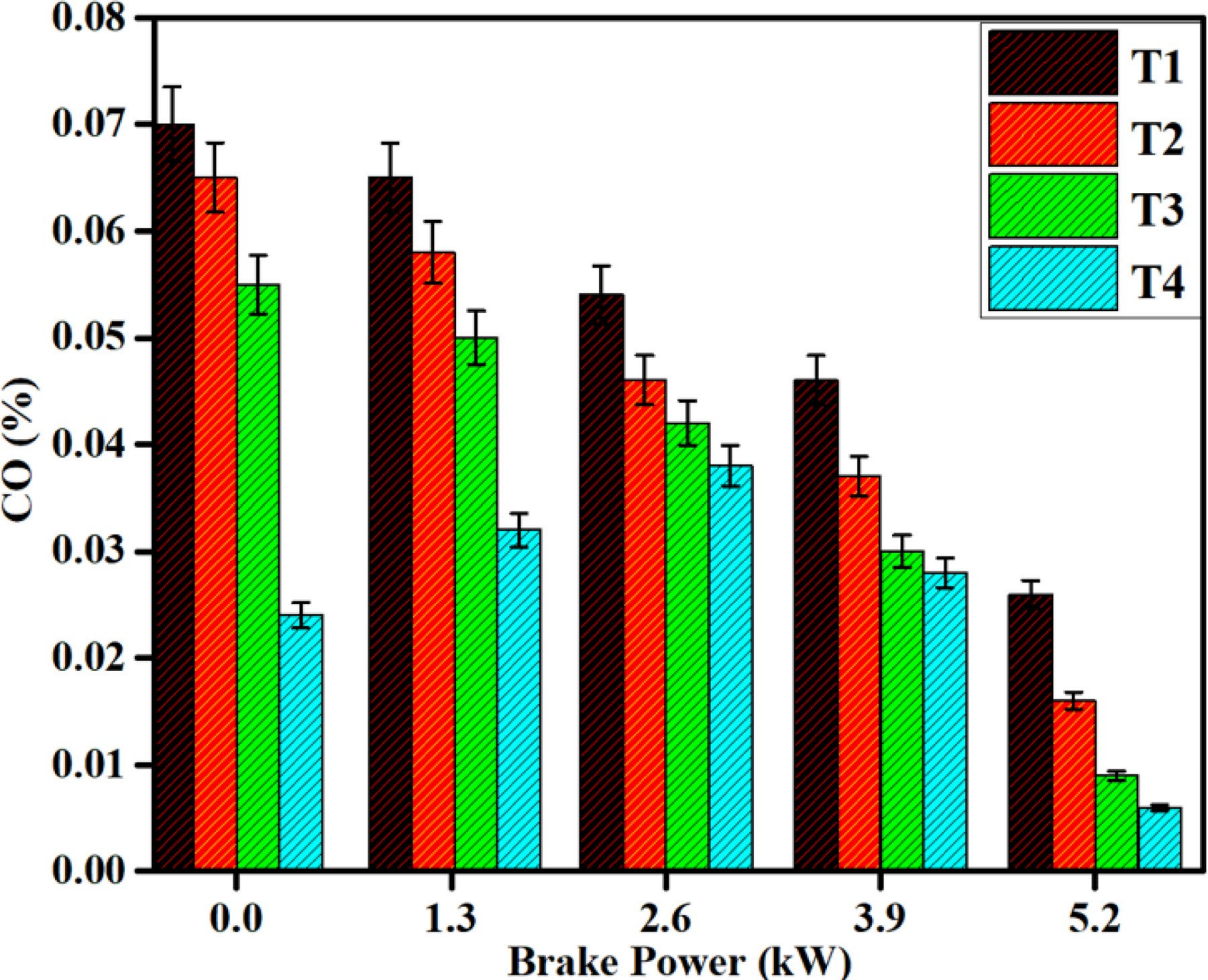
Difference in CO emissions with brake power for both diesel and DPB with and without a catalyst.

Figure 14 depicts the variation of CO emissions with brake power at the minimum and maximum loads. Diesel engines do not emit relatively large amounts of CO as they maintain a low equivalence ratio (f) coupled with high combustion temperatures. CO arises primarily through incomplete combustion processes. Furthermore, DPB has a higher ignition delay and shorter combustion duration than diesel fuel, due to its lower cetane number and higher aromatic compounds. Under all load conditions, an SCR after-treatment system is demonstrated to reduce CO emissions for both diesel and DPB fuels^41–43^. The use of an SCR system resulted in a 46% reduction in CO emissions for diesel fuel and a 50% reduction for DPB fuel at full load.

Resitgolu et al.^48^ reported that the ANCF catalyst led to a reduction of approximately 45% in CO emissions for the maximum load, although the synthesized catalyst Fe_2_O_3_–SiO_2_/Al_2_O_3_ reduced CO emissions by 53.45% CO conversion efficiency and was more efficient than the conventional ANCF catalyst. This enhancement in CO conversion was attributed to the catalysis ability of the synthesized Fe_2_O_3_–SiO_2_/Al_2_O_3_ catalyst to oxygen storage capability and redox behavior of properties to facilitate CO conversion to CO_2_.

The SiO_2_ and Al_2_O_3_ components serve to promote an extremely high surface area, while being quite stable at elevated temperatures, functioning as the supporting materials. These supports promote the dispersion of the Fe_2_O_3_ phase to efficiently function as a catalyst and will create well-defined active sites and improvements for CO oxidation. The Fe_2_O_3_-SiO_2_/Al_2_O_3_ catalyst works better because of its simpler composition more dispersed active components, synergistic space interactions, and better thermal stability. The overall product comprises a more effective and stable CO reduction catalyst than and a complex ANCF system.

#### Smoke emission

Using a combination of diesel and DPB fuels both with and without SCR demonstrates significant reductions in smoke emissions. The maximum smoke level increased to the highest load when using un-diluted plastic oil. Compared to diesel, DPB fuel shows a slight improvement in terms of smoke emissions. The lower combustion temperatures slower the flame propagation. Smoke and soot are produced in the combustion process. For diesel and DPB fuels, SCR after-treatment processes are a viable option to reduce smoke emissions, regardless of load. Smoke emissions were reduced by 55% and 60% with SCR for both diesel and DPB fuels. Resitgolu et al.^48^ have shown smoke emissions reduced by 52% when using ANCF. The exhaust main fold has a Fe_2_O_3_-SiO_2_/Al_2_O_3_ catalyst molded into it to mitigate smoke. Synthesis catalytic molds active sites interact with soot particles. The large surface areas and porous structures of the Al_2_O_3_ and SiO_2_ catalyst supports allow for successful diffusion among active sites. The Fe_2_O_3_ is effective oxidation catalysis and further mixed with SiO_2_ and Al_2_O_3_ the catalyst may perform better. This mixed catalyst may promote soot particles and HC emissions to oxidize thus reducing HC emissions^44,45^.

**Fig. 15:**
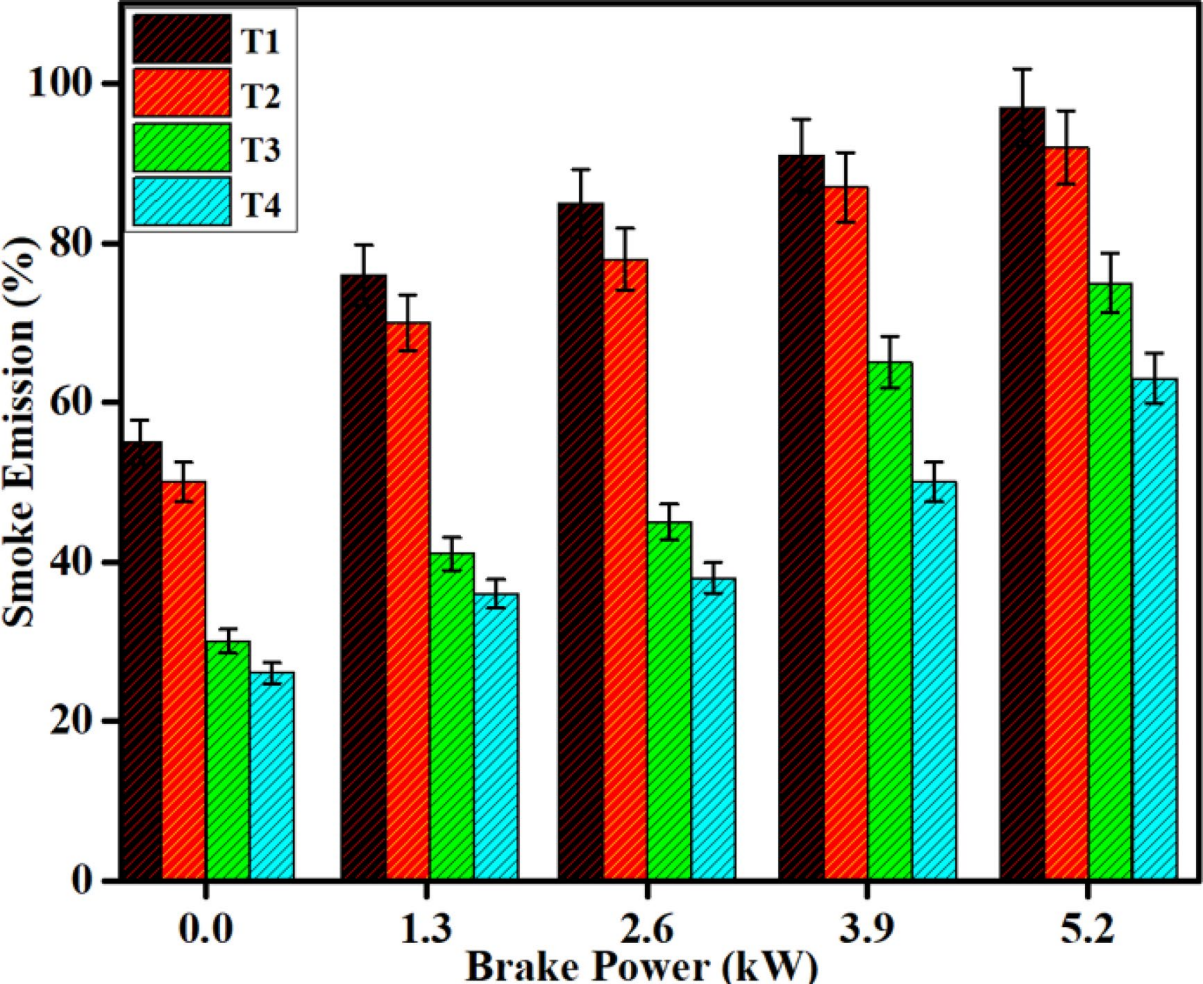
Difference in Smoke emissions with brake power for both diesel and DPB with and without a catalyst.

Fe_2_O_3_ and SiO_2_/Al_2_O_3_, when used together, can synergistically improve the overall catalytic activity. SiO_2_ helps to disperse the Fe_2_O_3_ particles while Al_2_O_3_ allows for a more robust structure and provides more active sites. Al_2_O_3_ is effective at preventing sintering at higher temperatures, which prevents the gathering of Fe_2_O_3_ particles and extends the catalyst’s lifespan. SiO_2_ and Al_2_O_3_ may also aid in protecting against catalyst poisons commonly found in diesel exhaust. ANFC is often correlated to a template utilizing Fe_2_O_3_-SiO_2_/Al_2_O_3_ as a catalyst. Catalysts such as Fe_2_O_3_-SiO_2_/Al_2_O_3_, with larger surface area, porosity, reactivity, and less likely deactivation, are typically more effective at reducing smoke emissions in diesel engines. All of these characteristics help to provide improved and more durable catalytic activity, which is an advantage when implemented within the smoke suppressor, as shown in Fig. 15, and also mass specific activity for pollutant reduction as shown in Table 10.

**Table 10:** Mass specific catalyst activity reduces pollutants under full capacity conditions.

Exhaust emission	Fuel	Total reduction (%)	Exhaust mass flow (g/h)	Catalyst mass (g)	Mass specific activity (g pollutant/g cat.h)
NO	DPB	85	4.2	500	0.0084
HC	DPB	53	1.4	500	0.0028
CO	DPB	50	5.4	500	0.0108
Smoke	DPB	60	0.28	500	0.00056

#### CO_2_ emission

**Fig. 16:**
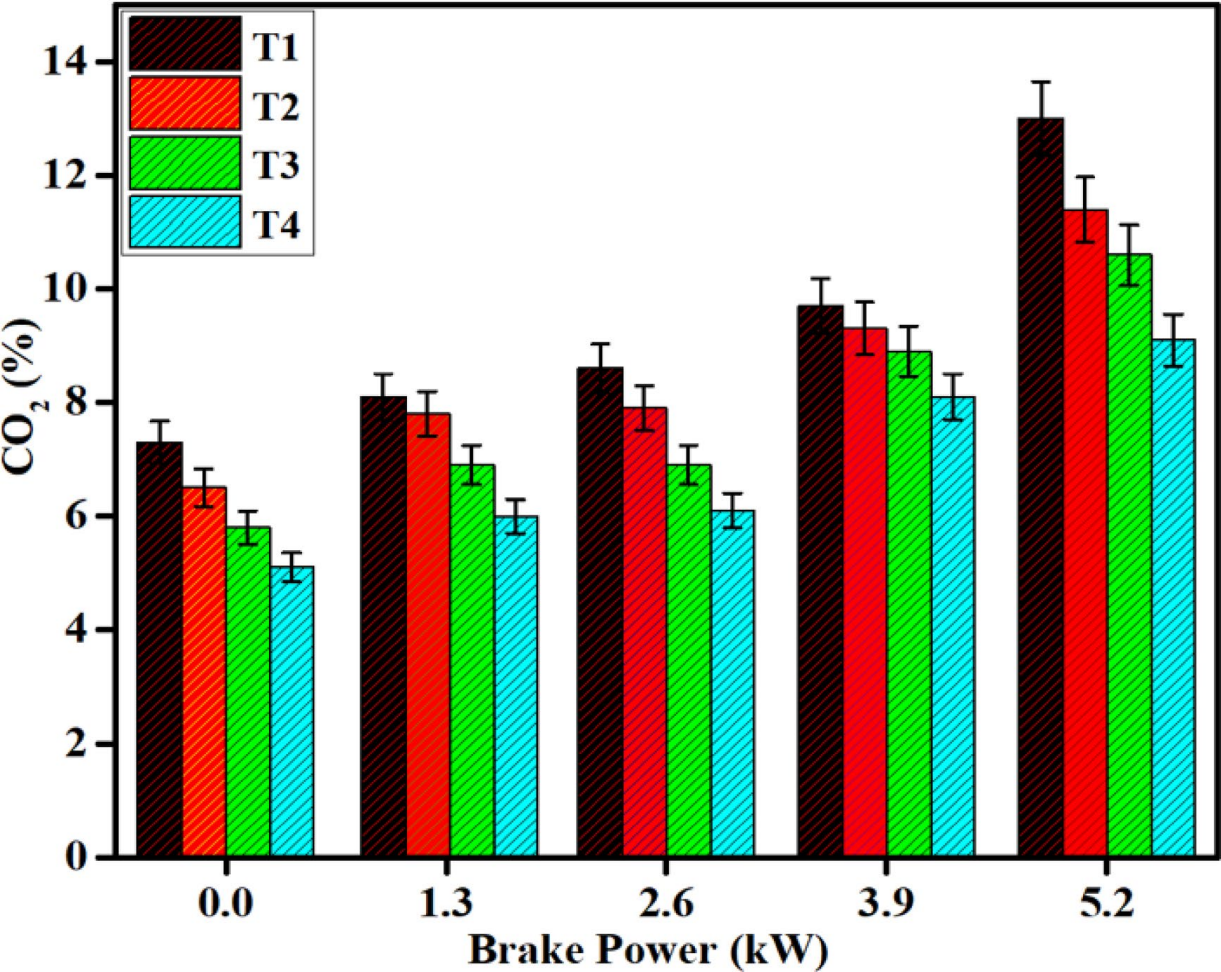
Difference in CO_2_ emissions with brake power for both diesel and DPB with and without a catalyst.

In Fig. 16 illustrates the variation of CO_2_ emissions with BP for diesel and DPB fuels, both before and after SCR catalyst installation. CO_2_ emissions are primarily associated with the extent of fuel oxidation during combustion and subsequent exhaust reactions. Therefore, CO_2_ concentration alone cannot be used as a sole indicator of combustion efficiency.

This study examined the amount of CO_2_ emitted by SCR-equipped engines alongside other emissions. The results indicate that SCR equipped engines produced lower levels of CO, HC, and smoke exhibited higher BTE levels; therefore, it can be inferred that SCR equipped engines maintain more complete combustion than engines without SCR equipment.CO_2_ emissions generated from post-combustion catalytic oxidation and changes to the overall exhaust gas mixture; therefore, CO_2_ emissions should not be interpreted as an identifier of poor combustion^46,47^.

The Fe_2_O_3_–SiO_2_/Al_2_O_3_ catalyst enhances the oxidation of residual CO and HC within the exhaust stream and maintains stable combustion in the cylinder. Al_2_O_3_ provides structural integrity and thermal stability to the exhaust stream when subjected to extreme Exhaust Gas Temperature (EGT); SiO_2_ helps to evenly distribute the active sites on Fe_2_O_3_ and prevent the agglomeration of catalyst particles. The use of a combination of SiO_2_, Al_2_O_3_ and Fe_2_O_3_ in this manner promotes increased catalytic activity without increasing the amount of incomplete combustion products.

CO_2_ emission trends as observed in this study represent the combined effect of fuel composition, exhaust after treatment reactions, and catalytic selectivity, and are not a direct measure of the combustion efficiency of SCR equipped engines. Therefore, combustion performance improvements will be evaluated based on NO, CO, HC, smoke and BTE levels, while CO_2_ emission trends will serve only as a secondary indicator of exhaust gas chemical make-up.

#### NO and NH_3_ conversion efficiency

**Fig. 17:**
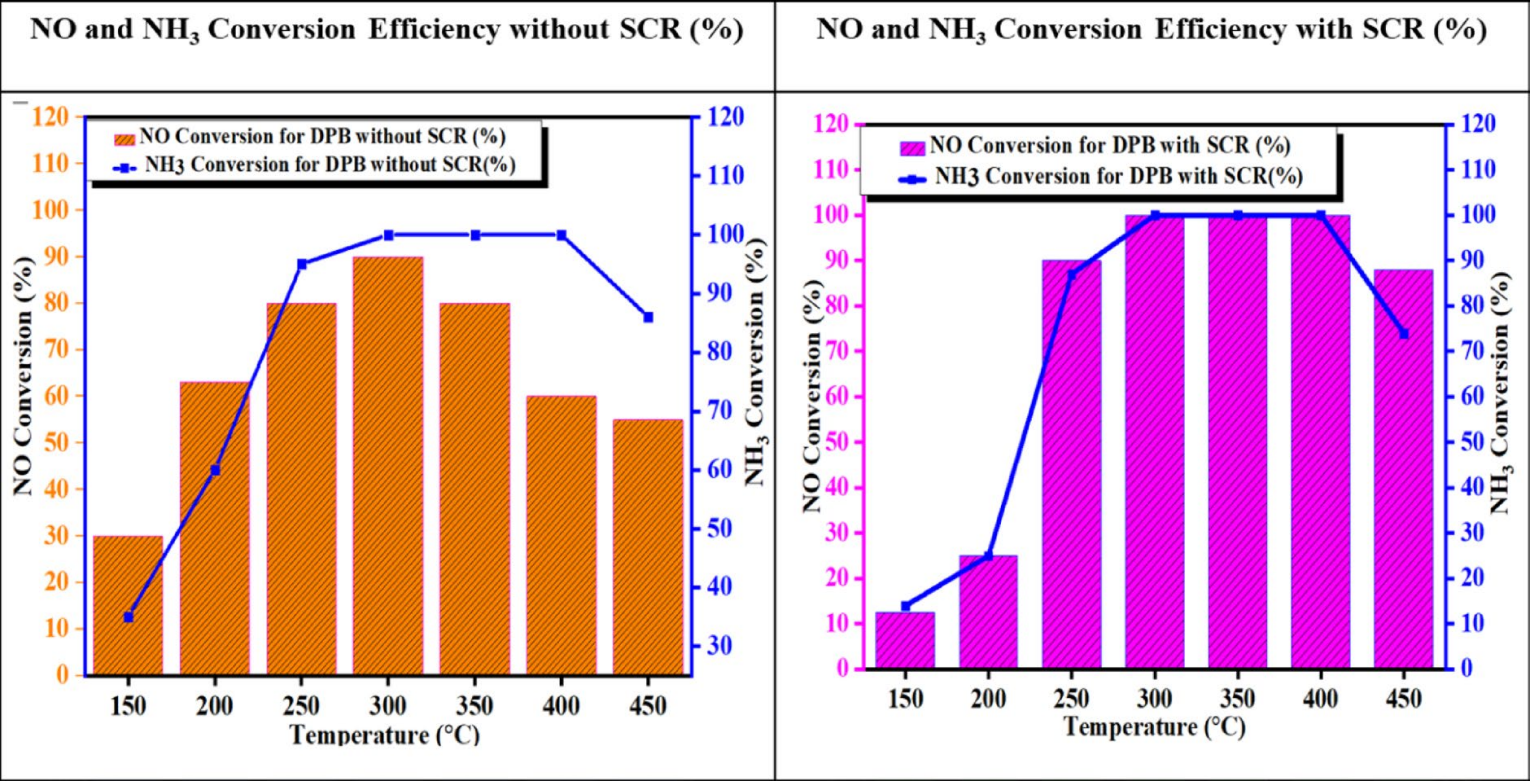
Temperature vs. NO and NH_3_ Conversion efficiency with and without SCR.

The graph demonstrates the activity of the Fe_2_O_3_–SiO_2_/Al_2_O_3_ catalyst in both the presence and absence of exhaust gas. At low temperatures (150 °C), the catalyst that has meet exhaust gas is less active than the one that has not encounter exhaust gas. At a higher temperature (approximately 300 °C), the catalyst exposed to exhaust gas outperformed the unexposed catalyst. The NH_3_-SCR system with the Fe–SiO_2_/Al_2_O_3_ catalyst can also achieve high conversion efficiency (> 85%) and good N_2_ selectivity due to the active sulfated iron added to the SiO_2_–Al_2_O_3_ support^49^. NH_3_ changes during the SCR reaction are noted. If the stoichiometric ratio between NO and NH_3_ conversion is maintained, the overall reaction efficiency increases. It is also important to mention that, at elevated temperatures, some degree of direct ammonia oxidation may also occur, which will mitigate the reduction of NO. The Fe_2_O_3_–SiO_2_/Al_2_O_3_ catalyst operates very efficiently in the sulfated SCR process at around 90 °C, as shown in Fig. 17.

#### HC and NH_3_ conversion efficiency

**Fig. 18:**
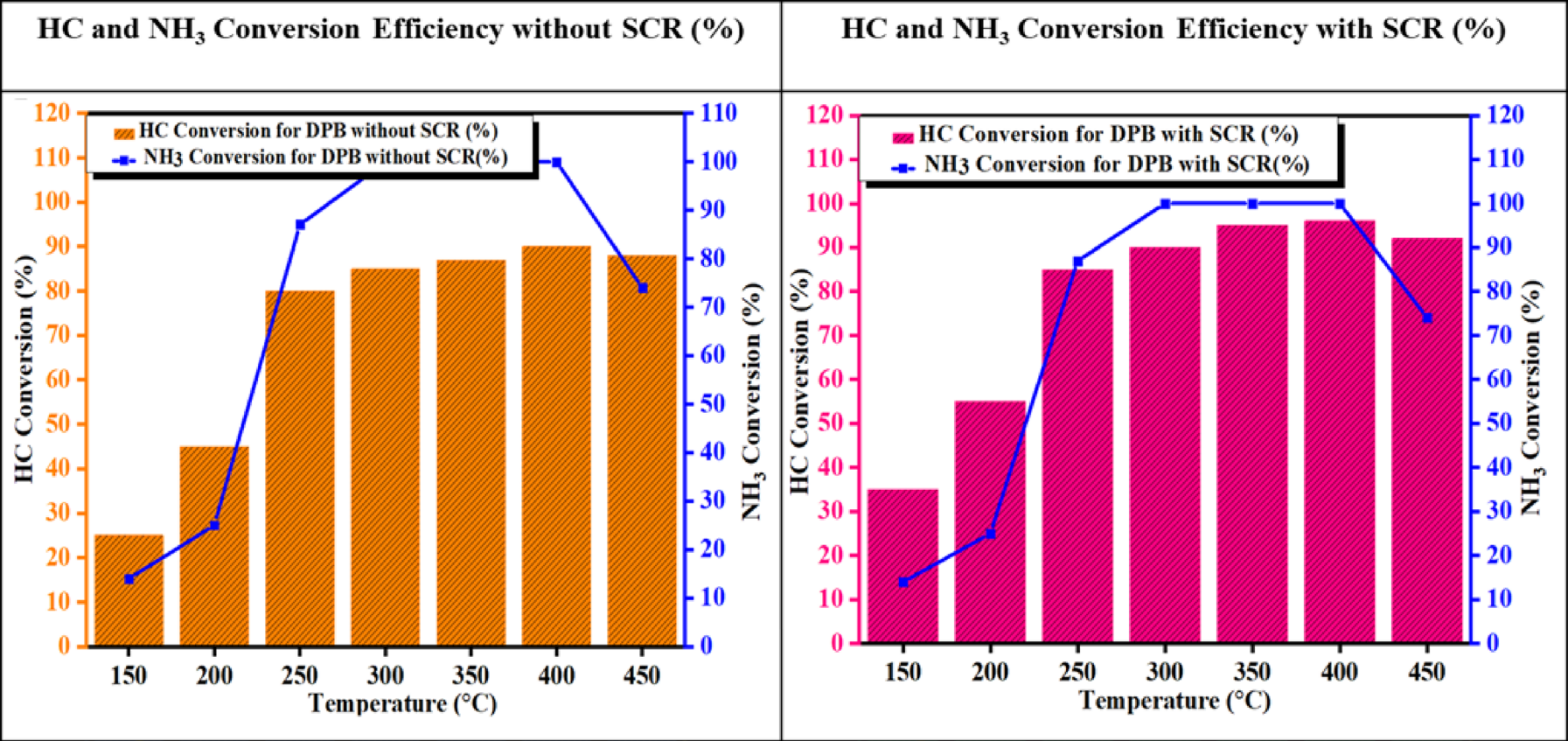
Temperature Dependence of HC and NH_3_ Conversion Efficiency with and without SCR System.

The increased HC conversion efficiency demonstrates that the NH_3_–SCR activity of the Fe–SiO_2_/Al_2_O_3_ catalyst is high compared to that of the Fe–SiO_2_/Al_2_O_3_ system, resulting from the synergistic interaction between the dispersed Fe species and SiO_2_/Al_2_O_3_. The increased specific surface area and number of uniformly dispersed active sites enhance effective adsorption and activation of both NH_3_ and NO; hence facilitating a selective pathway that ultimately generates N_2_ and H_2_O. The improved redox behavior of the Fe–SiO_2_/Al_2_O_3_ catalyst in Fig. 18 due to the fast Fe^3^⁺/Fe^2^⁺ cyclic transition keeps the oxidation–reduction equilibrium at the optimal level for the SCR reaction^49,50^.

The metal-SiO_2_/Al_2_O_3_ contact accommodates the stabilization of Fe Active Sites, leading to metallic sintering and deactivation at elevated temperatures. In addition to stabilizing the iron, the supporting structure provides additional mobility to lattice oxygen, creating conditions for catalyst turnover and reducing NO by-products. The synthesis of these materials exhibits significant selectivity towards N_2_ and H_2_O reduction products as well as efficient conversion rates. These findings indicate that the addition of iron results in improvement in the redox characteristics and distribution of active sites within the supporting SiO_2_/Al_2_O_3_ matrices, resulting in enhancement of the NH_3_-SCR catalytic reaction mechanism as well as reduction rates.

#### NH_3_ Slip and N_2_O emission considerations

NO direct measurements of NH_3_ slip formation were performed in this study, so the only data used to evaluate the SCR performance discussed in this article was based on the amount of NO converted, as well as the amount of HC, CO and smoke emissions reduced. Ammonia slips and N_2_O emissions are critical for properly assessing the performance of an SCR catalyst and the environmental effects of using it. Future work will involve monitoring NH_3_ and N_2_O continuously at various engine operating points to ensure that the reduction of NO occurs efficiently and does not result in unwanted secondary emissions. Through this approach, a complete evaluation of the selectivity and real-world applicability of the Fe_2_O_3_–SiO_2_/Al_2_O_3_ SCR catalyst has provided, as shown in Table 11.

**Table 11:** Comparison between NH_3_ slip and N_2_O formation.

Catalyst system	Typical NH_3_ slip (ppm)	N_2_O yield (%)	Temperature range (°C)
V_2_O_5_–WO_3_/TiO_2_	10–30	3–8	300–450
Fe-ZSM-5	5–20	1–4	200–500
Fe_2_O_3_–SiO_2_/Al_2_O_3_	< 25	< 2	150–600

#### CO and NH_3_ conversion efficiency

**Fig. 19:**
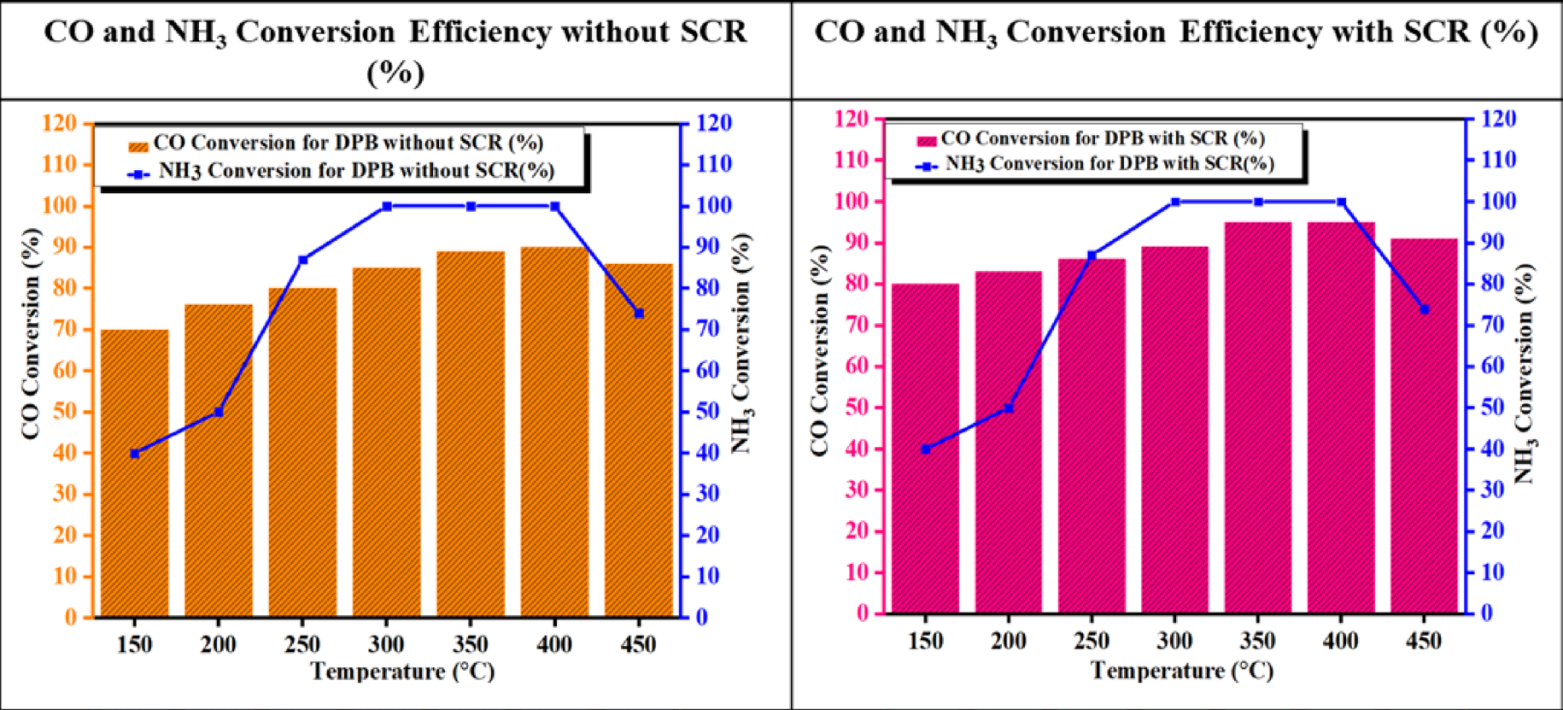
Temperature vs. CO and NH_3_ Conversion efficiency with and without SCR.

Research has shown that using Fe_2_O_3_—SiO_2_/Al_2_O_3_ as a synthesized catalyst results in a very high CO conversion efficiency in NH_3_—SCR. The large surface area contributes to reducing CO conversion, which also improves catalytic activity because the Fe particles are distributed throughout the large surface area. The extremely high density of active sites creates very high turnover rates and subsequent conversion efficiencies. Also, Fe naturally carries very good redox properties. Figure 19 shows great improvement for the conversion of both CO and NH_3_ to various products.

#### Advantages and difference between synthesis catalyst and commercial catalyst

The produced catalyst, with the combination of Fe_2_O_3_–SiO_2_/Al_2_O_3_ composition exhibits distinct advantages over typical commercial catalysts, particularly the PGMs which were not only scarce but also costly. The accessibility and lower price of alternate materials reduce the overall cost of the catalyst and potentially allow for larger-scale testing. The produced catalyst demonstrates significant reductions in NO, HC, CO, CO_2_and smoke, with total reductions of 80%, 74%, 60%, 65%, and 83%, respectively. It was also found to be more effective than a number of commercial compositions that were tested under similar conditions to this study.

High temperature conditions can adversely affect many commercially available SCR catalysts resulting in sulfate poisoning or thermal degradation of the synthesis catalyst. The commercial SCR catalyst used in this research project has shown to have thermal stability and oxidizing/reducing capabilities throughout the testing range of 150–600 °C which indicates the catalyst is suitable for practical application. Analysis of the catalyst using BET and SEM illustrates that the catalyst composed of a high surface area and proper porosity, thereby creating more opportunities for gas molecules to diffuse to an active site on the catalyst making it much more accessible and reactive than less favorable active sites. The combination of these silica and alumina supports provides better dispersion and interaction of Fe species within the catalyst system. Furthermore, through the combination of silica and alumina the catalyst creates a means for the storage of oxygen and to enhance both reactivity and thermal stability of the catalyst. In summary, by developing a new catalyst with enhanced catalytic activity, increased environmental sustainability for treatment options, and a cost-effective alternative to commercial systems provides us with multiple opportunities for commercialization. Table 4 (In Section “Advantages and difference between synthesis catalyst and commercial catalyst”) illustrates that the Fe_2_O_3_–SiO_2_/Al_2_O_3_catalyst provided the best NO reduction percentage (85%) compared to the V-based commercial catalyst (78.5%) and Zeolite catalyst (80%) respectively. Furthermore, the developed catalyst was able to provide stable catalytic activity at elevated temperatures (> 500 °C) while the vanadium catalyst experienced thermal deactivation, and the zeolite catalyst showed significantly lower hydrothermal stability than the Fe_2_O_3_–SiO_2_/Al_2_O_3_catalyst.

#### Durability, thermal stability, and structure–performance relationship

The research evaluated the durability of the Fe_2_O_3_–SiO_2_/Al_2_O_3_catalyst by analyzing its morphology both before and after exposure to diesel engine exhaust temperatures. The SEM images of the “fresh” catalyst demonstrate a uniform porosity throughout the catalyst and the presence of well-dispersed Fe_2_O_3_particles. The catalyst’s structural integrity was not impacted by diesel exhaust temperatures for a long time, with no major loss, such as sintering, agglomeration, or collapse of its porous structure.

The preservation of the mesoporous framework of the catalyst and stable dispersion of the Fe_2_O_3_particles within the SiO_2_/Al_2_O_3_ support contributes directly to its continued catalytic activity. As a result of the preserved structure and continued accessibility of the redox-active Fe^3^⁺/Fe^2^⁺ sites, the catalytic activity of this catalyst was maintained in engine operation, and the measurements taken using NO, HC and CO conversion efficiencies during engine operation confirmed the performance of the catalyst as a SCR catalyst for diesel exhaust after-treatment. The comparison of exhaust emission reduction performance of this work with that of previously developed SCR catalysts was shown in Table 12.

**Table 12:** Comparison of emission reduction performance of current Fe_2_O_3_–SiO_2_/Al_2_O_3_ catalyst versus previously reported SCR catalysts.

Types of catalyst	NO_x_ reduction (%)	HC reduction (%)	CO reduction (%)	Smoke reduction (%)	references
Fe_2_O_3_-SiO_2_/Al_2_O_3_	85	74	60	83	Present work
Fe_2_o_3_/Activated Carbon (Fe–Mn/AC)	97.5	–	83.3	–	^17^
Commercial V_2_O_5_-WO_3_/TiO_2_	80–95	–	–	–	^18^
Fe-Zeolite SCR catalyst	80	–	–	–	^19^
Cu-Zeolite SCR catalyst	90	–	–	–	^20^
Pt/Pd/Rh (PGM) catalyst	90–95	80–90	85–95	70–80	

#### Statistical analysis used in ANOVA

**Fig. 20:**
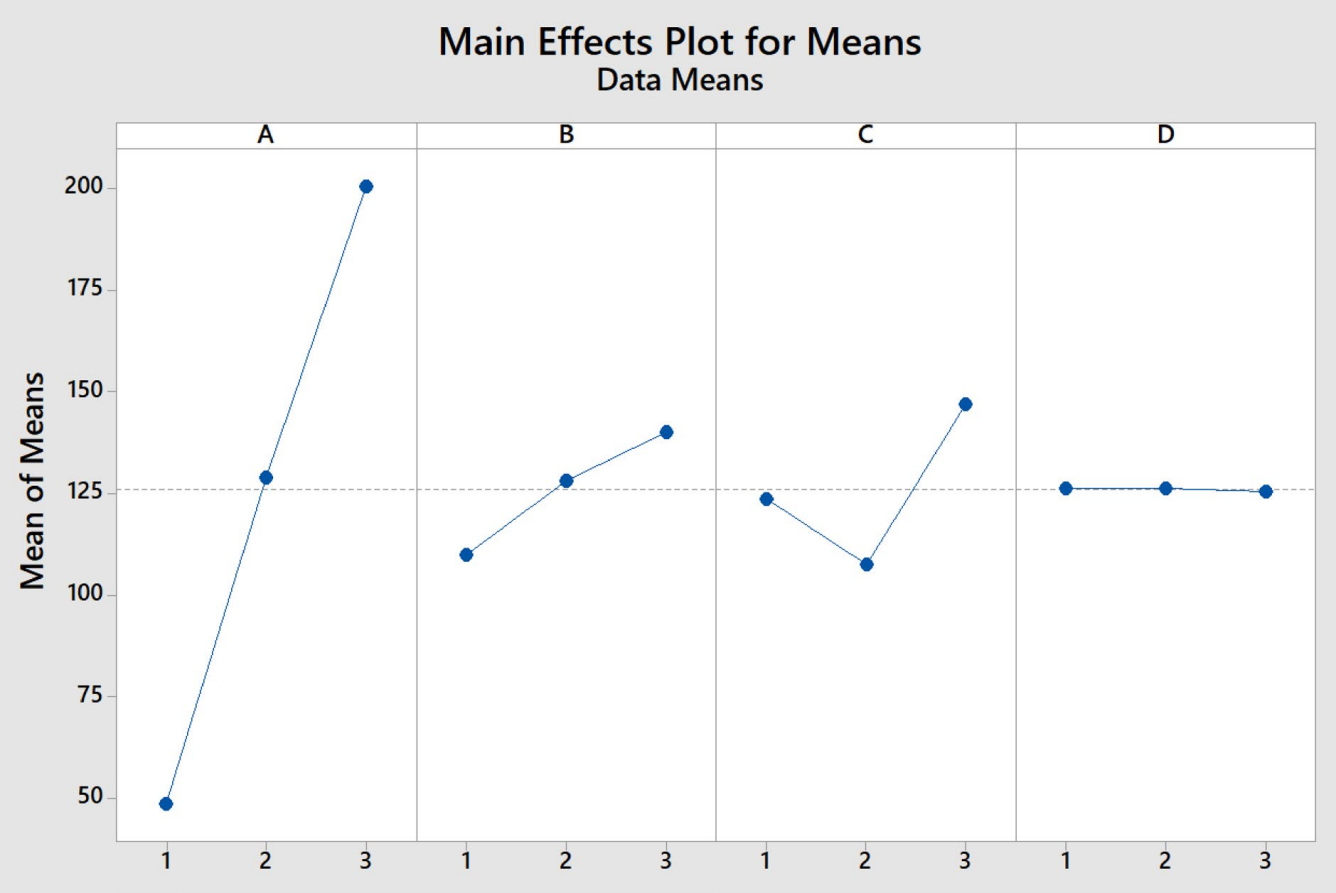
Means and data means are shown in exhaust emission.

**Fig. 21:**
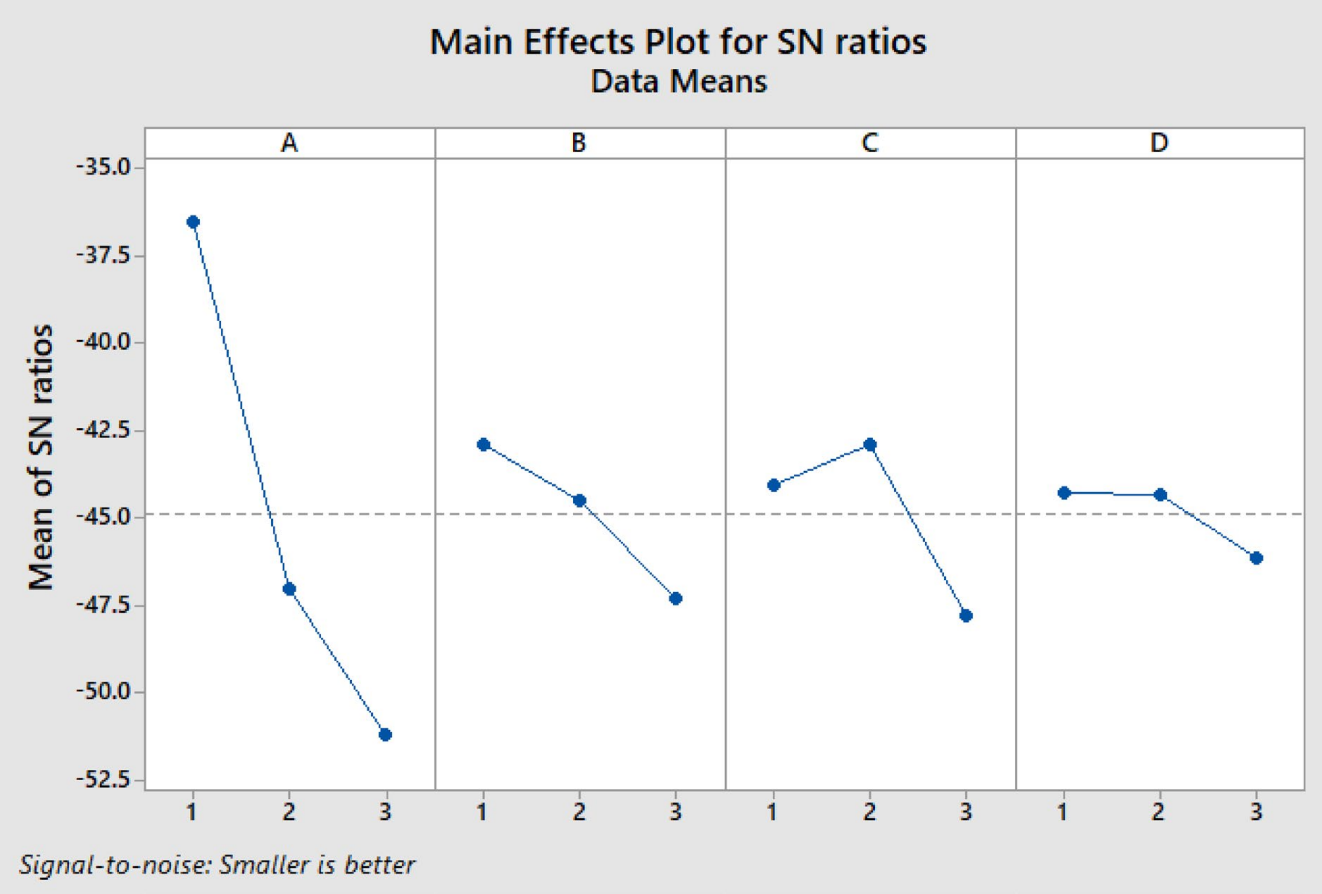
Main effects plot for SN ratios.

To statistically validate the experimental results, the ANOVA method was applied to the emission data obtained under different operating conditions. The ANOVA results presented in the table confirm that the variations in NO, HC, CO and smoke emissions were statistically significant, with p values below 0.05, indicating that the observed differences were not due to random experimental errors. The corresponding graphical trends illustrated in Figs. 20 and 21 further support s this statistical outcome, showing consistent and systematic emission reductions with the application of the Fe_2_O_3_–SiO_2_/Al_2_O_3_SCR catalyst. The close agreement between the ANOVA results (Tables 13 and 14) and the emission trends observed in figures confirm the reliability, repeatability, and significance of the experimental findings, demonstrating the effectiveness of the developed catalyst under real diesel engine operating conditions.

**Table 13:** SNRA values are calculated in exhaust emission.

A	B	C	D	NO	HC	CO	Smoke	SNRA 12
1	1	1	1	85	12	0.020	24	− 32.9803
1	2	2	2	90	15	0.024	26	− 33.5223
1	3	3	3	280	17	0.030	34	− 43.0019
2	1	2	3	320	20	0.034	36	− 44.1537
2	2	3	1	550	22	0.038	37	− 48.8132
2	3	1	2	500	025	0.025	39	− 47.9959
3	1	3	2	750	27	0.028	45	− 51.5018
3	2	1	3	720	29	0.015	50	− 51.1539
3	3	2	1	700	30	0.006	55	− 50.9160

**Table 14:** Percentage of contribution for input and output.

Source	DF	Adj SS	Percentage of contribution
A	2	331.107	88.91260916
B	2	37.158	9.978087842
C	2	2.788	0.748665399
D	2	1.343	0.360637601
Error	0	*	#VALUE!
Total	8	372.396	100

## Conclusion

A wide range of working-temperature catalysts was successfully developed as Fe_2_O_3_-SiO_2_/Al_2_O_3_ catalyst.

1. The integrated mold structure promotes strong Fe–O–Si, Fe–O–Al interactions, and uniform distribution of redox properties is active as Fe^3+^ /Fe^2+^ species and surface acidity are stable, which collectively enable efficient NH_3_ SCR activity over a wide range of temperatures, from 150 to 600 °C.
2. Engine scale evaluation on a diesel engine demonstrated a maximum NO reduction of approximately 85% and HC, CO and smoke emissions were also significantly reduced without adverse impact on BTE.
3. Mainly compared PGM and V_2_O_5_ based SCR techniques and then proposed Fe_2_O_3_–SiO_2_/Al_2_O_3_ catalyst offers a cost-effective, thermally stable, and environmentally benign alternative with wide operational flexibility.
4. The synthesis catalysts maintained structural and chemical integrity after prolonged exhaust exposure and exhibited consistent performance with both diesel and DPB.

These final results show the strong potential of the molded Fe-based SCR synthesis catalyst for scalable, sustainable after-treatment application.

## Data Availability

The datasets used and/or analysed during the current study are available from the corresponding author on reasonable request.

## Abbreviations

Al: Alumina
ANOVA: Analysis of variance
Al_2_O_3_: Aluminium oxide
ANCF: Al_2_O_3_–Nb_2_O_5_/CeO_2_/Fe_2_O_3_
ASTM: American society for testing and materials
BET: Brunauer–Emmett–Teller
BTE: Brake thermal efficiency
CC: Catalytic converters
Ce: Cerium
CeO_2_: Cerium (IV) oxide
CO: Carbon monoxide
CO_2_: Carbon dioxide
Cu: Copper
DOC: Diesel oxidation catalysts
DPB: Diesel plastic oil blend
DPF: Diesel particulate filters
EGT: Exhaust gas temperature
EPA: Environmental protection agency
EPR: Electron paramagnetic resonance
FTIR: Fourier-transform infrared spectroscopy
Fe^3+^: Ferric iron
Fe_2_O_3_: Iron oxide
HC: Hydrocarbon
H_2_: Dihydrogen
H_2_O: Water
KCl: Potassium chloride
K_2_CO_3_: Potassium carbonate
K_2_So_4_: Potassium sulphate
La: Lanthanum
LDVs: Light-duty vehicles
LNT: Lean NO trap
Mn: Manganese
Nd: Neodymium
NH_2_: Amino
NH_3_: Urea
NMR: Nuclear magnetic resonance
NO: Nitric oxide
NO_x_: Nitrogen oxides
NO_2_: Nitrogen dioxide
N_2_O: Nitrous oxide
Nb_2_O_5_: Niobium oxide
NH_2_NO: Nitrosamide species
O_2_: Dioxide
Pd: Palladium
PGM: Platinum group metals
PM: Particulate matter
Pt: Platinum
Rh: Rhodium
SCR: Selective catalytic reduction
SDG: Sustainable development goals
SEM: Scanning electron microscope
SiO_2_: Silicon oxide
TEM: Transmission electron microscopy
TPR: Temperature-programmed reduction
TPD: Temperature programmed desorption
TiO_2_: Titanium oxide
TEOS: Tri ethyl ortho silicate
V_2_O_5_: Vanadium oxide
WO_3_: Tungsten trioxide
XRD: X-ray diffraction
XPS: X-ray photoelectron spectroscopy
ZrSiO4: Zirconium silicate
